# Behavioral Impaired Control over Alcohol: Comparing Self-Medication and Behavioral Economic Models with the Interacting Effects of Acute Stress, Chronic Stress with Physical Neglect History, and Sex Assigned at Birth

**DOI:** 10.3390/bs16081342

**Published:** 2026-08-04

**Authors:** Julie A. Patock-Peckham, Jason Williams, Elena Kalina, Jessica R. Canning, Alondra Cruz, Ariana Ruof, Dylan C. Bauman, John D. Curtis, Danny Belton

**Affiliations:** 1 Department of Psychology, Arizona State University, 950 South McAllister, Tempe, AZ 85287, USA; amcruz11@asu.edu (A.C.); ariana.ruof@asu.edu (A.R.); dylan.bauman@asu.edu (D.C.B.); dbelton@asu.edu (D.B.); 2 RTI International, 3040 E. Cornwallis Rd, Research Triangle Park, Durham, NC 27709, USA; jawilliams@rti.org; 3 Department of Health Education and Behavior, University of Florida, 1908 Stadium Rd, Office 16B, Gainesville, FL 32611, USA; ekalina@ufl.edu; 4 Department of Psychiatry and Behavioral Sciences, University of Washington, 1959 NE Pacific St., Seattle, WA 98195, USA; jrcannin@uw.edu; 5 Department of Psychology, 1876 Campus Delivery, Colorado State University, Fort Collins, CO 850523, USA; jd.curtis@colostate.edu

**Keywords:** physical neglect, impaired control over alcohol, sex differences, stress, alcohol self-administration, ad libitum drinking session, trier social stress test

## Abstract

The Self-Medication Model predicts that individuals exposed to chronic stressful life histories (life experiences and physical neglect) will exhibit exaggerated behavioral impaired control over alcohol (i.e., IC; drinking beyond intended and/or recommended limits during ad libitum drinking) after experiencing acute stress. The Behavioral Economics Model predicts those with physical neglect histories (denied necessities) will be more likely to exhibit behavioral IC regardless of the presence or absence of acute and/or other chronic stress. Neither of these competing theories of alcohol addiction makes precise or distinct predictions concerning sex differences. We randomized groups of 2–3 drinkers (*N* = 210, 105 women, 105 men) to stress or no stress and alcohol or expectancy placebo conditions followed by a 90 min ad libitum social drinking session. We used the GLIMMIX procedure in SAS 9.4 to conduct a multilevel model for the number of standardized drinks ordered and Peak Blood Alcohol Concentration (BAC). We found significant three-way interactions of physical neglect level by stress condition by sex assigned at birth. Consistent with the Self-Medication Model, women with mean + 1 unit greater physical neglect histories showed higher behavioral IC only when the acute stress was present. In accordance with the Behavioral Economics Model, men with mean + 1 unit greater physical neglect histories showed higher behavioral IC when the acute stress was absent. Our competing theory test highlights the importance of physical neglect in alcohol misuse as well as the need to update etiological theories of addiction by accounting for sex differences.

“*Scientific revolutions occur when the current paradigm can no longer explain or accommodate anomalous observations within its framework*”

—Kuhn (2012)

## 1. Introduction

Alcohol is the leading risk factor for pre-mature death among adults aged 18–49 (GBD, 2018; Jernigan & Trangenstein, 2020). There are 28.9 million Americans who suffer from alcohol use disorder (AUD; SAMHSA, 2023), with 178,000 deaths annually (120,000 men; 59,000 women) making it one of the leading preventable causes of death in the United States (CDC, 2021; Pilar et al., 2020). Those who are experiencing AUD (1) become willing to incur costs of using alcohol that are both dysregulated and unacceptable to other people, and (2) participate in risky drinking (i.e., above legal limits) when given unrestricted access (Bickel et al., 2012). The National Institute on Alcohol Abuse and Alcoholism (2024) defines risky or heavy episodic drinking as five or more alcoholic drinks for men and four or more for women during a single drinking occasion, yet rarely do self-administration experiments examine levels beyond two to three standardized drinks (Patock-Peckham et al., 2022; see p. 3).

Few self-administration studies have samples of sufficient power to study sex differences (Patock-Peckham et al., 2022), so they fail to explain mechanisms driving AUD among the different sexes, with twice as many men dying each year (CDC, 2021). Men are also more likely to suffer from AUD (A. M. White, 2020), albeit this gap is closing for women (Peltier et al., 2019). Peltier et al. (2019) suggests these recent increases in AUD among women are due to early-life trauma combined with the need to escape current stressful feelings. Yet our current contemporary models of AUD fail to reliably predict distinct pathways and mechanisms that may be specific to sex assigned at birth under distinct situational contexts. To clarify the true etiology of the development of AUD, one must consider the contexts in which drinking occurs (Creswell et al., 2014; Sayette et al., 2016): individual trauma histories (Alvarez-Alonso et al., 2016; Davis et al., 2023; Patock-Peckham et al., 2020), genetics (Wagner et al., 2023; J. D. White & Bierut, 2023), individual differences in personality (Creswell, 2021; King et al., 2014), and sex assigned at birth (Keyes et al., 2010; Koob & White, 2017; McCaul et al., 2019; Peltier et al., 2019). While no single investigation can conceivably study all these things at once, we should strive to systematically fill these gaps in the existing literature.

We will first discuss different ways to measure drinking beyond limits and explain why we believe observed behavior in the moment may increase our theoretical understanding of the etiology of AUD. Next, we will discuss the need for critical tests to explore two distinct prominent and competing theories of AUD: (1) the Self-Medication Model and (2) the Behavioral Economics Model. We will compare these two distinct models by examining contextual factors that we have available and that are best able to move forward theory on dysregulated ad libitum drinking, such as acute stress, chronic stressful life histories (i.e., life experiences, physical neglect), as well as sex assigned at birth.

### 1.1. Ways to Measure Drinking Beyond Limits—Facets and Correlates

Consuming more alcohol than intended and for longer than intended best describes ***impaired control over alcohol use*** (IC; Chung & Martin, 2002; Heather et al., 1993; Leeman et al., 2012, 2014); it is one of the earliest indicators of AUD (Leeman et al., 2009, 2012, 2014; Vergés et al., 2021). Please see Table 2 in the methods of this current study for distinctions among types of IC. According to King et al.’s (2014) review, IC is most frequently examined with the Impaired Control Scale (ICS) part III (i.e., perceived belief in one’s inability to control one’s own drinking in the future; Heather et al., 1993). Perceived IC reflects a stable individual difference “*trait*” in cross-sectional survey work in emerging adulthood (King et al., 2014; Leeman et al., 2012, 2014; Patock-Peckham et al., 2022). Furthermore, perceived IC has been directly associated with self-control outcomes such as increased failures of self-regulation (Patock-Peckham et al., 2001, 2011). Belief in one’s perceived future inability to control one’s own drinking has also been directly associated with distinct alcohol use indictors and outcome variables such as increased alcohol use (Noudali et al., 2022; Patock-Peckham et al., 2018, 2020), heavy episodic drinking (Ebbert et al., 2018; Kalina et al., 2023), and alcohol-related problems and/or consequences (Bitsoih et al., 2023; Patock-Peckham et al., 2001, 2011, 2018).

Failed control in the past six months (ICS part II) longitudinally predicted increased alcohol-related problems over and above all Urgency–Premeditation–Perseverance–Sensation Seeking–Positive Urgency (UPPS-P; Cyders et al., 2007; Whiteside & Lynam, 2001) facets of impulsivity (Patock-Peckham & Corbin, 2023); this amplifies our need to systematically study IC. Notably, Vaughan et al. (2019) demonstrated that while in a solitary context, individuals with higher trait IC as measured with failed IC (Heather et al., 1993) achieved a higher blood alcohol concentration (BAC) during intravenous alcohol self-administration (IV-ASA). Despite cross-sectional correlational work on “*trait*” perceived future IC and a small handful of longitudinal and IV-ASA experimental work on failed control IC, there is clear lack of “*state*” (i.e., in the moment) behavioral IC experimental work during social drinking events. This gap in the literature renders improvements for therapeutic interventions and theoretical models stunted.

In this present study we seek to expand upon this existing literature on trait IC and study live dysregulated drinking with a newer behavioral method. In the moment, “*state*” **behavioral IC** represents an inability to stop or control alcohol use despite internal intentions and/or external incentives to limit consumption during ad libitum drinking (Leeman et al., 2013; Patock-Peckham et al., 2022). Behavioral IC reflects drinking more than the normative and intended one-to-three standardized drinks most social occasions dictate, leading to risky drinking as demonstrated in experimental ad libitum (free) drinking situations. Experimental studies regarding “*state*” (in the moment) behavioral IC using an ad libitum social drinking event with the individual’s preferred alcohol are rare; to our knowledge only two such published studies exist (Leeman et al., 2013; Patock-Peckham et al., 2022). As the intention to limit drinking is such a critical component of the distinction between impulsiveness and IC (Bickel & Marsch, 2001; Patock-Peckham & Corbin, 2023), experimental participants must be incentivized to limit their alcohol consumption.

Leeman et al. (2013) developed a novel behavioral IC paradigm utilizing individuals visiting a local bar who were given specific drinking guidelines (no more than three drinks in three hours) to model moderate alcohol consumption. Participants were instructed that if they over consumed and underperformed on cognitive tasks at the end of the night that they had a probabilistic chance of losing part of their subject payment. Those participants instructed to limit their consumption did drink more moderately than those not instructed to do so (Leeman et al., 2013). Therefore, it is possible to provide tangible incentives encouraging moderate drinking, conceivably affecting intentions to limit consumption.

Recently, Leeman et al.’s (2013) original paradigm was successfully modified to a 90 min ad libitum social drinking event with higher BAC cutoff levels (0.120) better reflecting true “*state*” behavioral IC (i.e., fast dysregulated risky drinking in a naturalistic bar laboratory) (Patock-Peckham et al., 2022). Patock-Peckham et al.’s (2022) study assessed the effects of acute stress on drinking in a social context demonstrating that cisgender women showed more behavioral IC when in the acute stress condition, but that cisgender men need a 0.065 BAC prime dose of alcohol as well as the acute stress to exhibit behavioral IC. Yet our original study failed to consider individual differences in chronic stressful life histories (i.e., life experiences and/or physical neglect), which is important to expanding the mechanisms behind our most prominent theories of AUD. Our purpose for this present study was to determine if distinct types of chronic life histories would interact with both experimental manipulations (acute stress) as well as sex assigned at birth during ad libitum drinking while controlling for alcohol prime condition. Next, we will discuss two theories of AUD that inspired this current set of investigators to examine multiple types of chronic stress in conjunction with acute stress: (1) Self-Medication, and (2) Behavioral Economics.

### 1.2. Self-Medication Model: Acute and Chronic Stress

The Self-Medication Model (Conger, 1956; Hersh & Hussong, 2009; Khantzian, 1997) predicts that those individuals exposed to acute stress (i.e., having to give a speech and perform math in front of an audience) will seek to quell their negative emotions with increased alcohol consumption. Experiments (Amlung & MacKillop, 2014; Ramchandani et al., 2018) involving comparisons of dependent and non-dependent drinkers show that both the subjective value of alcohol as well as craving for alcohol increase after a stress challenge. Acute stress is well known to increase both alcohol craving and alcohol consumption using a two-drink taste test paradigm (Sinha, 2009). We build off Sinha’s (2009) work on understanding normative acceptable social drinking behavior to explain the mechanisms involved in risky heavy episodic drinking by testing which individuals engage in behavioral IC (Patock-Peckham et al., 2022) despite an increased probability of consequences.

Experiments with a psychosocial stressor such as the Trier Social Stress Test (TSST; Kirschbaum et al., 1993) have shown at least that non-dependent social drinkers voluntarily consume more alcohol immediately after exposure to the acute stress manipulation (de Wit et al., 2003; Magrys & Olmstead, 2015; McGrath et al., 2016). Recent experiments (Patock-Peckham et al., 2022, p. 8) have shown that the TSST robustly increased heart rate among participants versus controls. Furthermore, meta-analyses have shown the TSST to reliably produce a physiological stress response such as a spike in cortisol among healthy individuals (Goodman et al., 2017; Helminen et al., 2021). Thus, we predicted that both cisgender men and women would drink more after the TSST stress manipulation even with the inclusion of other chronic stressful life history variables. Nevertheless, an unpublished meta-analysis from Germany encompassing 44 mostly underpowered experimental studies with 77.5% male participants found that the impact of acute stress on alcohol consumption will diminish with adjustment for small study effects (Weckesser et al., 2025) adding to the controversy of the role of sex assigned at birth and its role on alcohol self-administration. Therefore, we strongly felt our larger sample of both men and women may clear up some of this controversy despite a lack of clear prediction regarding the variable of sex assigned at birth.

The Self-Medication Model also predicts those with overall more chronically stressful life experiences will be more likely to consume excessive amounts of alcohol when available. Stressful life experiences (e.g., terrorism, natural disasters, divorce, job loss, maltreatment) have long been reported by those with AUDs as a reason for individuals using alcohol (Keyes et al., 2011). Chronic stress in particular has been implicated as a vulnerability to addiction (Sinha, 2008, 2024), and sudden traumatic life events have also been associated with relapses for those in treatment for AUD (Sinha, 2022). Chronic stressful life events in childhood pose a 1.27 increased risk for 9–10-year-old children to begin sipping alcohol (Nagata et al., 2023). Negative life events were also associated with an increased odds of alcohol use initiation among 12–21-year-old adolescents (Zhao et al., 2023). Interestingly, Chaplin et al. (2018) proposed that chronic stress in childhood leads to sex differences in reactivity to acute stressors such that girls experienced increased reactivity, while boys experienced blunted reactivity. Yet, to our knowledge, stressful life events before the age of 12 and acute (in the moment) stress are rarely studied simultaneously in the same alcohol self-administration investigation to examine which is the determining mechanism or if they are additive in nature. Chronic stress is an important assumption of the Self-Medication Model for AUD and is frequently measured as a negative life event index (e.g., Life Experiences Survey; LES; Johnson & McCutcheon, 1980), but our original (Patock-Peckham et al., 2022) experiment failed to include it as a potential moderating variable. Thus, we hoped to build upon Sinha’s (2009) seminal work by including both a measure of negative life event history measured prior to our drinking event as well as an acute stress manipulation on the day of our drinking experiment.

### 1.3. Physical Neglect and AUD

One type of chronic stress not captured in the Life Events Survey (Johnson & McCutcheon, 1980) is physical neglect, so we will study both chronic stress through life experiences as well as physical neglect as potential independent moderators in our experiment. Physical neglect is a form of childhood maltreatment that involves denying children proper access to food, clean clothing, suitable shelter, and medical attention by caregivers; these children regularly reside in unstable environments under the care of unreliable people (Bernstein et al., 2003). On any given day in the United States, one in five children (18.5%) under the age of twelve report physical neglect at the hands of their caregivers (Finkelhor et al., 2015).

Individuals with alcohol addiction (Alvarez-Alonso et al., 2016) and comorbid disorders often report severe childhood maltreatment (Cullins et al., 2025). Due to the still developing brain, abuse experiences in early childhood (before the age of 12) are more detrimental than other traumas broadly defined and experienced later in life (Bellis, 2001; Wang et al., 2023; Yu et al., 2024). Given the critical implications of childhood maltreatment, including physical neglect, on dysregulated drinking behavior, it is critical to systematically examine this relationship in an experimental drinking study. To our knowledge, we will be the first study to ever investigate behavioral IC with physical neglect as a potential moderating factor.

Rubio et al. (2025) found that heavy drinkers and alcohol-dependent individuals were more likely to have experienced physical neglect as young children. Furthermore, longitudinal research found that early physical neglect exposure prior to the age of 12 poses an increased probability for the subsequent development of internalizing and externalizing symptoms and riskier alcohol use behaviors among emerging (Cohen et al., 2017) and middle-aged adults (Widom et al., 2015). Davis et al. (2023) found individuals who reported higher levels of physical neglect were more likely to show rapid longitudinal growth in alcohol use when followed from ages 17–24 years. Individuals with a history of physical neglect are at greater risk for earlier onset of alcohol use (Dube et al., 2006; Poznyak & Rekve, 2018) and show faster increases in heavy episodic drinking during adolescence and emerging adulthood compared to those without a history of physical neglect (Shin et al., 2013). Thus, physical neglect is an important variable to consider in the development of risky drinking behavior among emerging adults. It is possible that physical neglect is a unique form of chronic stress that is critical in the formation of AUD.

Cross-sectional studies have found direct associations between physical neglect histories prior to the age of twelve and perceived IC (Heather et al., 1993) over and above other trauma facets (i.e., lack of support, emotional, physical, and sexual abuses; Bitsoih et al., 2023; Noudali et al., 2022; Patock-Peckham et al., 2020). Noudali et al. (2022) established that physical neglect was directly associated with perceived IC over and above other trauma facets, and insomnia symptoms. In addition, Bitsoih et al. (2023) found physical neglect was directly associated with perceived IC over and above other trauma facets, as well as coping motives for drinking. Furthermore, Patock-Peckham et al. (2020) demonstrated that over and above post-traumatic stress disorder (PTSD) symptoms and all other facets of trauma, physical neglect was directly associated with perceived IC; this pattern was slightly stronger for men. However, survey ratings of perceived future control over alcohol do not always translate into actual behavior and these studies were not experimental in nature. These studies do however suggest that physical neglect is directly associated with perceived IC and that physical neglect may more strongly predict the behavior of cisgender men than women (Patock-Peckham et al., 2020).

According to Davis et al. (2023), few studies have considered disparities in rates of physical neglect as a predictor of alcohol use based on potential moderators such as sex assigned at birth and other demographics. This is especially true in the context of alcohol self-administration studies which are often grossly underpowered to study sex assigned at birth as a variable or fail to even study women at all (Patock-Peckham et al., 2022; see p. 3). The systemic underrepresentation of women in these studies has left major gaps in our understanding of behavioral IC, limiting theoretical advancements and, ultimately, the development of targeted AUD interventions. By incorporating both physical neglect history and sex assigned at birth into experimental designs, particularly in ecologically valid social drinking contexts, we can help bridge these gaps and refine models of IC in AUD. Utilizing investigations well suited to study sex differences is important because as children, males across many cultures are more likely to experience physical neglect than are their female counterparts (Dong et al., 2024; Egry et al., 2015; Sobsey et al., 1997). Men are also more likely to suffer from AUD than are women (A. M. White, 2020). This implies that studying both physical neglect and sex assigned at birth in the same ad libitum social drinking event study may be rather useful for improving our existing theories on alcohol addiction.

### 1.4. Behavioral Economics Model and the Role of Physical Neglect

Seminal researchers from the Behavioral Economics Model have theorized that physically neglected individuals are especially more prone to overindulging in reinforcing substances such as food and alcohol (Bickel et al., 2012; Bickel & Marsch, 2001; Vuchinich & Heather, 2003). Under this model, individuals prone to AUDs often prefer smaller more immediate unhealthy rewards (e.g., another drink) over larger more distal, healthier rewards such as a clear mind the next day at work (i.e., delayed discounting; Acuff et al., 2021; Amlung & MacKillop, 2014; Gaume et al., 2022). We define **behavioral IC** as choosing the immediate reward of another drink right now versus the uncertain long-term reward of a better subject payment at the end of a drinking event (Leeman et al., 2013; Patock-Peckham et al., 2022). As such, behavioral IC is a representation of delayed discounting with alcohol as the reward.

Along with Bickel et al.’s (2012) seminal writings on the topic, Vuchinich and Heather (2003) also suggested potential moderators for accelerating behavioral IC such as limited access to critical necessities so common among the physically neglected. Reck et al. (2024) showed that household food insecurity during adolescence longitudinally affected neural systems such as resting state functional connectivity as well as impulsive behaviors and choices. Merely imagining the scarcity or uncertainty of monetary and social resources increases rates of delayed discounting (Bickel et al., 2017; Sze et al., 2017). Delayed rewards are inherently unreliable (Takahashi et al., 2007) and this uncertainty increases probabilistic discounting (Mazur, 1993) especially among those with childhood maltreatment histories (Oshri et al., 2018a, 2018b). Thus, as the probability of the delayed reward decreases, the probability of selecting the smaller immediate more reliable reward increases (Vanderveldt et al., 2015). Yeh et al. (2021) suggests that delayed discounting may be an individual characteristic and/or trait on its own.

Increased uncertainty around current and future events increases discounting of delayed rewards (Acuff et al., 2021). For instance, Snider et al. (2020) showed individuals who had experienced a natural disaster who were now in a state of physical neglect without their normal access to shelter, food, or clothing experienced greater discounting compared to controls; immediate rewards and survival were prioritized. Conceivably, physically neglected individuals will take all the reinforcing items they can including alcohol when available because it may not be reliably available again in the future (Bitsoih et al., 2023). Based on the Behavioral Economics Model of Addiction (Acuff et al., 2021; Amlung & MacKillop, 2014; Bickel et al., 2012; Gaume et al., 2022), those who had been physically neglected may be less likely to follow such instructions to limit alcohol consumption as they are more likely to overconsume reinforcing substances when available regardless of the negative consequences.

Behavioral Economics suggests that physical neglect history is a risk factor for behavioral IC, yet it does not make any predictions concerning the effects of acute and/or other forms of chronic life stress on these relationships. The Behavioral Economics Model predicts that those scoring higher in physical neglect history would demonstrate more behavioral IC during ad libitum drinking than those scoring lower in physical neglect history regardless of their acute stress condition or sex assigned at birth. We gleaned our predictions about sex assigned at birth from a recent cross-sectional study suggesting cisgender men may be slightly more affected by physical neglect than cisgender women when this concerns perceived IC (Patock-Peckham et al., 2020), but this was an exploratory hypothesis concerning behavioral IC.

### 1.5. Summary of Hypotheses

We set up our study as a test of two competing theories of AUD: (1) the Self-Medication Model versus (2) the Behavioral Economics Model. Based on the Self-Medication Model, we expected that those participants in our stress (TSST) condition would exhibit more behavioral IC overall than those in the no stress (no TSST) condition even with the inclusion of chronic stress variables in our models, with effects being more pronounced for women. Further, according to the Self-Medication Model, we expected that those with higher negative life event histories would exhibit more behavioral IC than those with lower negative life event histories. Moreover, if the Self-Medication Model is more explanatory, we expected those with a mean + l units of physical neglect history would exhibit the greatest degree of behavioral IC when acute stress was present; thus, we expected these factors to be additive in nature. Conversely, if the Behavioral Economics Model was more explanatory, then individuals scoring higher in physical neglect history should exhibit the greatest degree of behavioral IC during the social ad libitum drinking session regardless of their acute stress condition or sex assigned at birth. We did not have clear predictions regarding either competing theory regarding sex assigned at birth. Instead, we drew upon Chaplin et al.’s (2018) study that suggests that women will be more reactive to chronic stress life histories via increased negative emotions. Further, we drew on Patock-Peckham et al. (2020) which suggested that cisgender men may be slightly more affected by their physical neglect history than cisgender women. Regardless, our large sample size (*N* = 200+) afforded us the ability to examine the effects of potential moderators (i.e., acute stress, sex assigned at birth) with physical neglect and/or life events history in distinct tests concerning actions in a group drinking session to explain “*state*” behavioral IC.

## 2. Methods

### 2.1. Participants

We recruited 221 participants via flyers posted around the community as well as around the main and downtown university campuses. We excluded those with a previous medical history contradicting the use of alcohol (i.e., anti-depressant and/or psychotropic drugs). Eligible participants were between 21 and 35 years of age (*M* = 23.05; *SD* = 2.87); they also reported drinking at least three alcoholic beverages over a 2 h period at least once in the last month. Next, to screen out alcohol dependence and other psychiatric disorders, all participants were screened with a full Alcohol Use Disorder and Associated Disabilities Interview Schedule (AUDADIS; Grant & Dawson, 2000) battery approximately two weeks prior to their bar lab appointment. In addition, on the day of their bar lab appointment, we also screened all female participants with a home pregnancy kit. Ten participants were excluded for one of three reasons: (1) due to sessions with scheduled participant no-shows leading to some solitary drinking appointments, (2) the participants clearly stated to our staff that they had zero intention of drinking that evening, and (3) because they were more than 1.5 standard deviations above or below the target BAC within the prime condition (>0.085 or <0.046). Our final sample consisted of 210 individuals (*n* = 105 women, *n* = 105 men). Our sample consisted of the following ethnic categories: 25% Hispanic/Latino and 75% Non-Hispanic. Our sample consisted of the following racial categories: 72% White, 11% Asian, 8% African American, 4% Native American, 1% Pacific Islander, and 4% other, respectively. Sociodemographic data and average drinking habits in the past 30 days can be found in Table 1.

The distinct types of IC assessments discussed in the existing literature versus the behavioral IC method we utilized in this current experiment are summarized in Table 2.

### 2.2. Measures

We collected pre-screening measures before the social drinking session portion of the experiment. Pre-screening sessions were on average held two weeks before the bar lab session. During this 2–3 h mental health pre-screening session we collected negative stressful life events and physical neglect histories. These distinct chronic stress indicators were used as potential moderators along with our acute stress condition (TSST, No TSST), and sex assigned at birth (women, men). The alcohol prime dose condition (0.065 BAC cocktails, 0.000 BAC expectancy cocktails) pre ad libitum drinking was used as a covariate due to no clear theoretical predictions as well as with the aim of providing models simple enough to obtain proper standard error bars and point estimates for our multi-level models tested in SAS 9.4.

### 2.3. Pre-Screening Measures for Chronic Stress Used as Potential Predictors/Moderators

We measured two types of chronic stress prior to the social drinking event. There were zero overlapping items on the negative life events and physical neglect measures described below.

#### 2.3.1. Negative Life Events

We utilized the Life Experiences Survey (LES; Johnson & McCutcheon, 1980) containing a 46-item self-report measure regarding a wide range of stressful life events such as experiencing your parent’s divorce, job losses, arrests, moving, and serious illnesses in close family members. It also includes negative personal life events such as being robbed or mugged, serious illness or injury, failing to make a sports team, and/or being bullied. We used the sum score for this measure as our indicator of negative life events. Due to the wide range of phenomena covered in this measure, the Cronbach α reliability was 0.67. For reference, Cronbach’s alpha reflects an index of a unidimensional construct; life events are more multidimensional in nature than an average scale. Nevertheless, this measure is widely understood as a marker for accumulated unrelated stressful events (Sarason et al., 1978).

#### 2.3.2. Physical Neglect Prior to the Age of Twelve

The Childhood Trauma Questionnaire (CTQ; Bernstein et al., 2003) consists of 25 items that measure emotional abuse, physical abuse, sexual abuse, physical neglect, and emotionally supportive family members from childhood prior to the age of 12. Physical neglect was defined as the “failure of caretakers to provide for a child’s basic physical needs, including food, water, shelter, clothing, safety, and health care” (Bernstein et al., 2003). We only utilized the physical neglect items for this study. Physical neglect consists of 5 items (Bernstein et al., 1994, 2003), including “I wore dirty clothing”, “there was not enough to eat”, “I was not taken to a doctor”, “I was not taken care of” and “my parents were drunk or high”. Responses were on a 5-point scale in which responses included *never true, rarely true, sometimes true, often true, and very often true* (Bernstein et al., 1994, 2003). The Cronbach α reliability for physical neglect was 0.71.

### 2.4. Dependent Variables and Predictors

The behavioral IC method we utilized in our current experiment is defined in Table 2. As a proxy for behavioral IC or dysregulated risky drinking we utilized two distinct continuous outcome variables during the 90 minutes ad libitum drinking session: (1) the number of standard drinks ordered, and (2) peak BAC. The focal predictors of the two drinking outcomes were the stress condition and sex assigned at birth with types of chronic lifetime stress histories (i.e., physical neglect or life events) which were measured prior to the group bar session.

### 2.5. Covariates

Based on the literature, we controlled for several potentially relevant covariates in our models. We controlled for (1) usual drinking quantity, (2) anxiety sensitivity, (3) the experimental alcohol priming dose condition, and (4) BAC at the start of ad libitum drinking (for models with peak BAC as the outcome).

#### 2.5.1. Usual Drinking Quantity

Using a single item (Wood et al., 1992), which asked, “What is your usual quantity of alcoholic beverages consumed at one drinking occasion?” for one drink equivalent to one bottle or can of beer, one glass of wine, or one drink of distilled spirits. The usual quantity of alcohol consumed in one drinking occasion was coded 1 = 1 drink, 2 = 2 drinks, 3 = 3–4 drinks, 4 = 5–6 drinks, 5 = 7 or more drinks. This measure was collected at the pre-screening lab session approximately two weeks before the bar lab session.

#### 2.5.2. Anxiety Sensitivity Index

(ASI; Reiss et al., 1986) is a 16-item scale that measures an individual’s concerns about negative consequences associated with anxiety-related symptoms (e.g., “It scares me when I feel faint,” “It is important to me to not appear nervous,” and “It embarrasses me when my stomach growls”). Response options included 1 = very little, 2 = a little, 3 = some, 4 = much, and 5 = very much. The Cronbach α reliability was 0.87 for this sample. ASI was collected at the pre-screening lab session.

#### 2.5.3. Alcohol Priming Dose Condition

We included the alcohol prime dose condition (0.000 BAC, 0.065 BAC) as a covariate rather than an additional moderator after models examining the impact of the priming experimental manipulation indicated that the associations of interest between sex assigned at birth, acute stress, and chronic stress on drinking outcomes were not conditional on the priming condition. Those randomly assigned to the alcohol expectancy condition, in which participants received 3 cocktails of cranberry juice, 7-up, and flattened tonic water achieving a BAC of (0.00) prior to ad libitum drinking, we coded as zero. Those randomly assigned to the alcohol condition, achieving an average BAC of 0.065 prior to ad libitum drinking and received 3 cocktails based on their weight and cisgender sex consisting of cranberry juice, 7-up, and vodka, we coded as one.

#### 2.5.4. BAC Before Ad Libitum Drinking Period

Each participant’s reading for BAC at the start of ad libitum drinking session was used to covary initial BAC from peak BAC to assess change due to the predictors of interest during ad libitum drinking (Allison, 1990). Initial BAC was measured just prior to the ad libitum drinking event and was not included in models with the number of drink orders as the outcome.

### 2.6. Measures Utilized for Our Manipulation Checks

We used the following manipulation checks: (1) heart rate measured seven times using Garmin wrist watches, and (2) a measure of state anxiety. Heart rates were measured “at baseline”, “after relaxation breathing”, “after we announced the speech”, “after the speech preparation”, “after the videotaped speech”, “after the speech review form”, and “after the survey measuring state anxiety”. We measured state anxiety with a very short 2–3 minutes survey immediately following the speech and the math task or the control group task. The State-Trait Anxiety Inventory (STAI; Spielberger et al., 1983) is a 20-item measure of in-the-moment anxiety and includes items asking participants if they felt tense, worried, nervous, upset, etc., right now in the moment. The Cronbach α reliability was 0.93 for our sample.

### 2.7. Experimental Manipulations

Our groups of 2–3 participants were taken into individual rooms and separated from their cohorts during both parts of the TSST and control tasks, although they did all their drinking together in the bar lab. All drinking groups of 2–3 people were randomly assigned to the same stress and alcohol prime condition.

#### 2.7.1. Trier Social Stress Test (TSST)

To make the TSST (Kirschbaum et al., 1993) more socially evaluative and threatening, both the speech and math portions of the TSST were given in front of three experimenters trained to express only non-reinforcing, flat, and dismissive facial expressions. We used two timed and video-taped sessions. Part I required that the participants take 5 minutes by themselves to prepare. Part II required participants to give a 5 minute speech about their likes and dislikes regarding their own personality, appearance, and life in front of an audience. The following instructions were provided for all participants:

“Please recall that your speech is on ‘WHAT YOU LIKE AND DISLIKE ABOUT YOUR APPEARANCE, PERSONALITY, AND LIFE’. Your speech will be videotaped and reviewed by a team of evaluators who have been carefully trained to evaluate you for positive and negative statements, openness, defensiveness, and several other psychological factors. We will not be able to talk or answer any of your questions during your speech. You may begin.”

Part III of the TSST is an arithmetic task during which the participant must verbally count backwards from 1022 in increments of 13 for 5 minutes. Participant errors during the math task required individuals to start over from 1022. Participants in this stress (TSST) condition were videotaped during both the speech and the math tasks. In summary, the stress (TSSST) condition consisted of 15 minutes of tasks: Part I—5 minutes to prepare, Part II—5 minutes to give the speech, and Part III—5 minutes of doing math. Parts II and III were videotaped.

#### 2.7.2. No Stress (No TSST) Control Condition

This task required participants to rate the videotaped speech of one of our research confederates posing as a previous subject. For Part I, all participants in the no stress (no TSST) condition received the following instructions:

“In approximately 5 minutes from now you will be required to evaluate a 5-minute speech from a previous participant. The topic of the speech was ‘WHAT YOU LIKE AND DISLIKE ABOUT YOUR APPEARANCE, PERSONALITY, AND LIFE.’ Please spend the next five minutes writing about what characteristics you feel are important for people who are giving presentations to be evaluated on. We are interested in your input.”

During Part II, we announced the video (traits/characteristics people should be evaluated on):

“You may use a sheet of paper and pen to write down your thoughts in the next five minutes. Please leave your watch on, we will take a reading after the 5 minutes elapses. We have prepared our own speech evaluation form that we are hoping to improve with your thoughts and the thoughts of other participants. However, at this time, we would like you to view this video of another participant giving a speech on ‘WHAT HE LIKES AND DISLIKES ABOUT HIS APPEARANCE, PERSONALITY AND LIFE.’ Do you have any questions?” [Answer Questions] “Please just carefully watch this video for the next 5 minutes.”

The experimenter then prepared a 5 minute timer and read the following instructions:

“Please evaluate this speech for positive and negative statements and check it for openness, defensiveness, and several other psychological factors. You will have five minutes to fill out this form. If you finish early, there is an additional comments section where you can write about how well you thought this participant performed based on the evaluation criteria you wrote about earlier”.

Part III of the control condition consisted of a 5 minute task to serve as a counterpart control to the second part of the TSST (the 5 minute math task). We asked our participants to write about what characteristics they felt were important for people who are giving speeches to be evaluated on in our future experiments emphasizing that we were very interested in the participants input to improve our criteria for how speeches would be evaluated in the future. In summary, the no stress (no TSST) control condition consisted of 15 minutes of tasks: Part I—5 minutes to think about characteristics of speeches, Part II—5 minutes to view our confederate’s video-taped speech, and Part III—5 minutes to evaluate the confederate’s speech and to provide feedback for our future experiments.

### 2.8. Procedures

All participants arrived at the lab at approximately noon on Friday or Saturday. Participants were randomly assigned in groups of 2–3 to a stress condition [stress (TSST), no stress (no TSST)] and a beverage condition [a priming dose of alcohol 0.065 target; an expectancy placebo 0.000]. Alcoholic beverages were a cocktail of cranberry juice, sprite, and vodka (calculated by sex assigned at birth/height/weight; see Curtin & Fairchild, 2003; BAC calculator). Control condition beverages were a cocktail of cranberry juice, sprite, and flattened tonic water with the glasses rimmed with a negligible amount of alcohol for taste and smell cues. All study staff bartenders were blind to the prime dose beverage condition. The IRB approved the protocol with a peak BAC of 0.120. At the start of the protocol, participants completed baseline measures on a battery of computerized cognitive tasks which were used as part of our cover story. Then, depending upon the stress condition, participants completed either the TSST or control task and then all participants completed the 90-minute ad libitum drinking session, followed by a subset of the computerized cognitive tasks again.

After the stress condition manipulation, the alcohol administration period began. We served participants either alcohol or placebo cranberry juice-mixed beverages at a rate of one drink every six minutes with a one-minute break between each drink, followed by an eight-minute absorption period. Post the alcohol administration period, participants completed a survey and another subset of cognitive tasks. Next, participants began the 90-minute ad libitum drinking period and were instructed to limit consumption with the below language.

#### 2.8.1. Violation of Impaired Control Instructions

“Early this evening you completed several tasks. Later, you will repeat some of these tasks. It is very important that your performance is consistent both times you complete these tasks. If your performance on the second task falls below 80% of your performance on the baseline measure, you will be required to draw from a hat with one of three statements: (1) no loss, (2) a loss of $10.00, (3) or a loss of $20.00. Thus, if you must draw from the hat, you will have a 33% probability of losing your full $20.00 bonus payment. Remember, you have consumed alcohol this evening and alcohol does affect your performance on these tasks. It is recommended for optimal performance that you do not consume more than one standard drink per hour”.

Each alcoholic beverage registers approximately 0.020 BAC. Presumably given that most people can excrete and metabolize approximately 0.015 BAC per hour, limiting intake to one drink per hour should produce relatively minor cognitive impairment on the cognitive tasks and avoid any chance of a reduction in subject payment. The experimenter then recorded each time the participant ordered another drink as well as each time they wanted another drink but were not served due to hitting the BAC limit of 0.120.

#### 2.8.2. Ad Libitum Drinking Period

The ad libitum drinking session comprised a 90-minute free social drinking period in which participants could order alcoholic or non-alcohol drinks from our bar menu and self-administer freely until either a BAC of 0.120 was reached, or they voluntarily decided to stop. Once a BAC of 0.120 was reached, participants were barred from ordering any additional alcoholic beverages for the duration of their time in the session. We used a BAC calculator from the University of Wisconsin (Curtin & Fairchild, 2003) to determine if a particular participant should be barred from ordering additional alcoholic beverages. Breathalyzer tests were only taken during the ad libitum drinking session if a participant was likely to exceed a BAC of 0.120; otherwise, drinking was uninterrupted. A ride home was provided after the protocol was completed and participant’s BAC reached 0.020 or less. Participants were paid $12 dollars per hour plus a $20 dollar bonus for their performance on cognitive tasks performed after the ad libitum drinking session. Participants were provided with food, Netflix, and board games while waiting for their BACs to return to 0.020 or below.

### 2.9. Data Analytic Strategy

Each group of participants was randomly assigned to the same experimental condition (stress or prime). To account for clustering in the data, we utilized a generalized linear mixed model (GLMM, also called multilevel models (MLMs) or hierarchical linear models (HLMs)) to account for the groups of 2–3 people in a naturalistic bar lab drinking context. Analyses were performed in SAS 9.4 (SAS Institute Inc., 2025; Cary, NC, USA) using the PROC GLIMMIX procedure and a Gaussian response distribution for continuous outcomes.

We conducted two distinct models of stress histories to examine physical neglect and life events histories as potential moderating variables. First, we conducted a GLMM with a stress condition (TSST, No TSST Control) by sex assigned at birth (women, men) by **physical neglect** history with the alcohol prime condition, life events, anxiety sensitivity, and usual drinking quantity as covariates; see Table 3. Second, we conducted a GLMM with a stress condition by sex assigned at birth by **life events** with the alcohol prime condition, physical neglect, anxiety sensitivity, and usual drinking quantity as covariates; see Table 4. We utilized BAC at the start of ad libitum drinking session as an additional covariate for models concerning Peak BAC as the outcome variable. All non-binary variables were centered to facilitate main effect interpretation in the context of interactions. Model parameters were used to generate estimates and statistical tests of simple main effects and lower order interactions from the full models. To probe our significant 3-way interactions we conducted simple effects tests comparing mean levels versus mean + 1 unit greater for the chronic stressor (physical neglect or negative life events) included in the model. The comparisons facilitated exploration of sex-assigned-at-birth differences concerning standardized alcoholic drink orders made and peak BACs reached during the 90-minute ad libitum drinking period. We also used the same models to estimate the overall effects for all items in the interactions.

## 3. Results

Our calculations yielded ICCs of 0.36 for the number of standardized drinks ordered and 0.47 for peak BAC during the 90 minute ad libitum drinking session. These values indicate between 36% to 47% of the variance in drinking outcomes is attributable to group-level differences and the remaining variance is attributable to within-person differences; this highlighted the need to use MLM to examine within-person differences in drinking outcomes (Hox, 1998), while accounting for the social drinking context.

### 3.1. Manipulation Checks

We examined manipulation checks to decide if our stress (TSST) manipulation was effective in our study. Participants assigned to the stress (TSST) condition showed an increased heart rate compared to those in the no stress (no TSST) condition. Those in the stress (TSST) condition had significantly higher heart rates than controls after the speech announcement, F(1, 206) = 14.33, *p* < 0.001 *** as well as after the video-taped speech, F(1, 206) = 37.66, *p* < 0.001 ***. Furthermore, participants in the stress (TSST) condition (*M* = 35.35) reported a significantly higher state anxiety than those in the no stress (no TSST) condition (*M* = 27.33), F(1, 209) = 42.37, *p* < 0.001 ***.

### 3.2. Results for Dependent Variables

#### 3.2.1. Model: Physical Neglect as a Predictor and Life Events as a Covariate for Drink Orders

**Figure 1:**
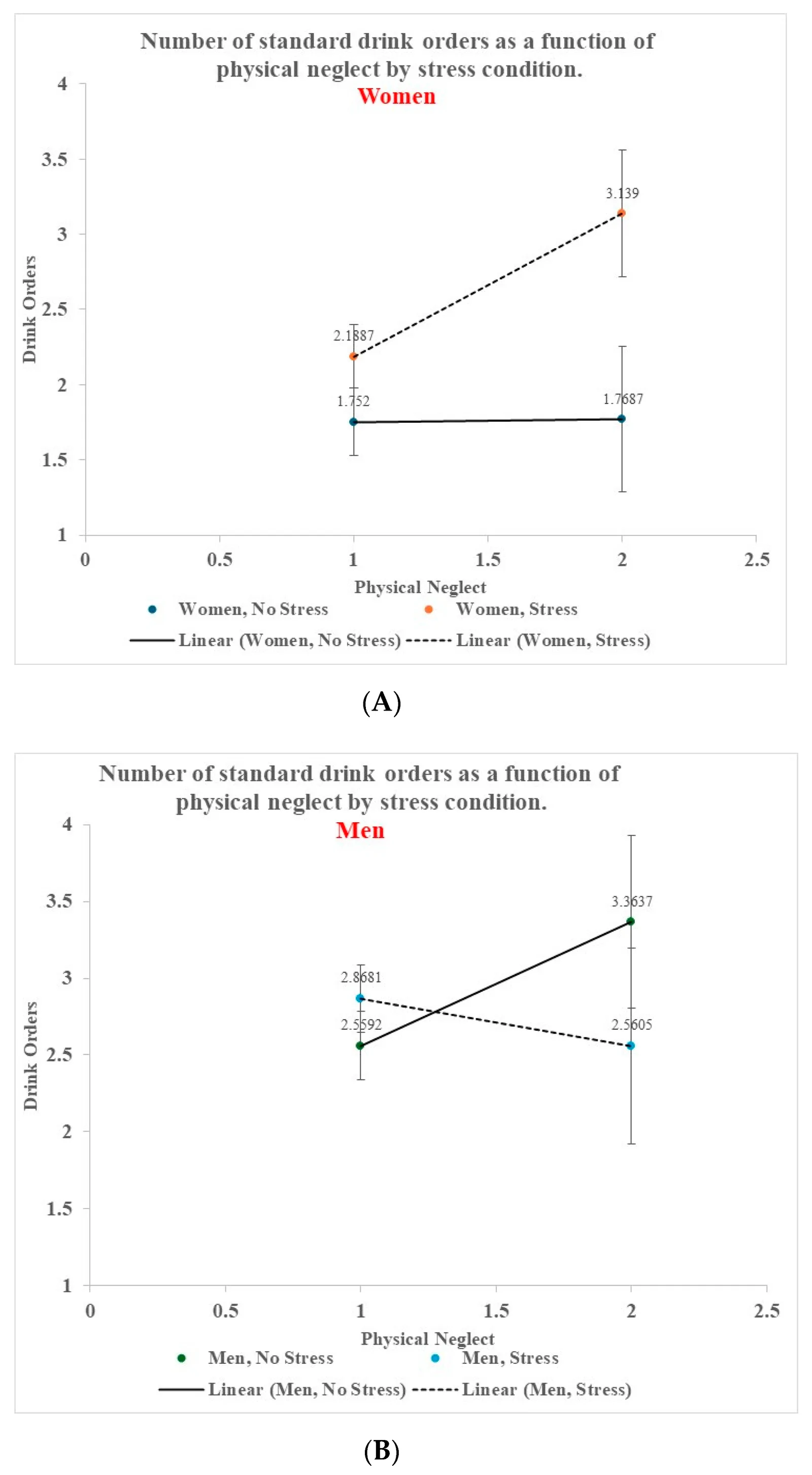
(**A**,**B**) Number of standard drink orders as a function of stress (TSST, no TSST) condition by level of physical neglect history by sex assigned at birth (women, men).

We found a significant overall main effect (using comparisons of the least squares means) of sex assigned at birth for standardized drink orders. Overall, men ordered about 0.8 more standardized drinks [(2.6 drinks); β = 0.743, S.E. 0.174, t (1, 90) = 4.27, *p* < 0.001 ***] than did women (1.8 drinks). The overall main effect of physical neglect accounting for all other main and interaction effects was not significant (β = 0.377, S.E. 0.243, t (1, 90) = 1.55, ns). The overall main effect of stress condition was similarly nonsignificant (β = 0.375, S.E. 0.209, t (1, 90) = 1.80, ns). The main effect of sex assigned at birth was modified by a significant three-way interaction of stress condition by sex assigned at birth by physical neglect history, β = −2.046, S.E. = 0.955; t (1, 90) = −2.14, *p* < 0.035 *. This significant three-way interaction is depicted in Figure 1A,B. All other main and lower-order interaction effects included in the model can be found in Table 3. All main effects and interaction terms tested in the GLMM are shown in the models presented in Table 3 and Table 4. It is worth noting that Table 3 and Table 4 account for the GLMM regression parameters while accounting for all other effects in the model including the interactions. The reader may find more conventionally interpreted main effects reflecting overall or omnibus tests for each variable (i.e., marginal overall main effects vs. model parameter main effects that represent single simple main effects at 0 levels of other factors in the interaction) in Table 5. We discuss those values when discussing main effects in the body of the manuscript. Simple effects for the probed interactions and the point estimates we used to create the error bars can also be found in Table 5.

#### 3.2.2. Simple Effects of Physical Neglect for Standardized Alcoholic Drink Orders

We conducted simple effects tests to probe the three-way interaction depicted in Figure 1A,B, showing the interaction of stress condition by sex by physical neglect history for standardized alcoholic drink orders. The figures display four simple effects for the sex- and stress-specific physical neglect effects (the lines in the plots) as well as four simple effects for the effect of stress condition differences by sex and at mean neglect and +1 unit greater physical neglect (point estimates with error bars). We also estimated the simple effects for differences in drink orders by sex assigned at birth at levels of physical neglect and by stress condition, but these were not easily discerned from the plots.

##### Simple Effects for Physical Neglect (See the Left Side of Table 5)

Higher physical neglect values significantly increased the number of standardized drink orders only for women in the stress (TSST) condition (β = 0.950, S.E. = 0.376, t (1, 90) = 2.53, *p* = 0.013 *). Women with mean +1 unit greater physical neglect histories ordered (3.14) drinks while those with mean level of physical neglect ordered (2.19) drinks. Women in the no stress condition showed little change associated with increased physical neglect (β = 0.017, S.E. = 0.428, t (1, 90) = 0.04, *p* = 0.969). 

This pattern did not replicate for men. Men showed nonsignificant effects of physical neglect on drink orders in opposing directions [stress condition: β = −0.308, S.E. = 0.574, t (1, 90) = −0.54, *p* = 0.593; no stress condition: β = 0.805, S.E. = 0.501, t (1, 90) = 1.61, *p* = 0.111]; this is shown in Figure 1B where two lines can be seen going in the opposite direction.

##### Simple Effects for Stress Condition (See the Left Side of Table 5)

There was a single significant simple effect of stress condition on drink orders among women with elevated physical neglect. Women at +1 unit greater physical neglect in the stress condition ordered significantly more alcoholic drinks during ad libitum drinking (3.14) than those in the no stress condition (1.77); β = 1.370, S.E. = 0.612, t (1, 90) = 2.24, *p* = 0.028 *. Stress condition did not significantly impact drink orders in women at the mean level of physical neglect (β = 0.437, S.E. = 0.270, t (1, 90) = 1.62, *p* = 0.110). 

Men showed no significant effect of stress condition at +1 unit physical neglect (β = −0.803, S.E. = 0.838, t (1, 90) = −0.96, *p* = 0.340) or at mean level of neglect (β = 0.309, S.E. = 0.272, t (1, 90) = 1.14, *p* = 0.259).

##### Simple Effects for Sex Differences (See Left Side of Table 5)

Simple effects of sex assigned at birth indicated increases in the number of drink orders for most combinations of stress and values of physical neglect. In the no stress condition, there was a significant simple effect for sex assigned at birth at mean levels of physical neglect, β = 0.807, S.E. = 0.252, t (1, 90) = 3.20, *p* = 0.002 ***; men ordered more alcoholic drinks (2.56) than did women (1.75). Moreover, for the no stress condition at mean +1 unit greater physical neglect concerning sex assigned at birth, there was a significant simple effect, β = 1.595, S.E. = 0.722, t (1, 90) = 2.21, *p* = 0.030 *; men ordered more drinks (3.36) than did women (1.77).

In the stress condition there was a significant simple effect for sex assigned at birth at mean levels of physical neglect, β = 0.679, S.E. = 0.238, t (1, 90) = 2.85, *p* = 0.005 **; men ordered more standardized alcoholic drinks (2.87) than did women (2.19). Significant sex differences in drink orders (2.56 for men, 3.13 for women) were not found for the stress condition at +1 unit greater physical neglect (β = −0.579, S.E. = 0.743, t (1, 90) = −0.78, ns).

#### 3.2.3. Model: Physical Neglect as a Predictor and Life Events as a Covariate for Peak BAC

**Figure 2:**
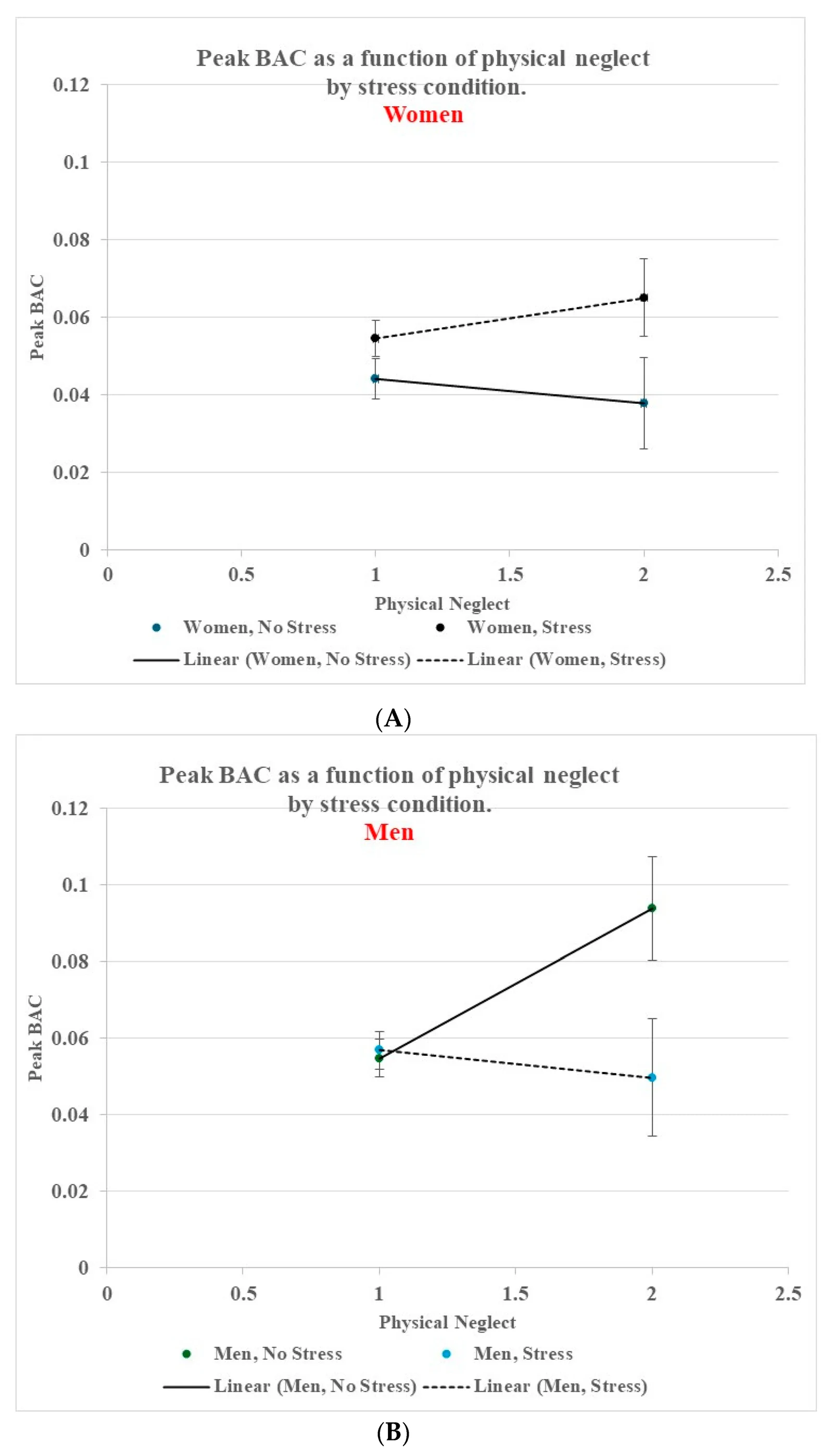
(**A**,**B**) Peak BAC reached during the 90 min social free drinking period as a function of stress condition (TSST, no TSST) by physical neglect history by sex assigned at birth.

The models with peak BAC and physical neglect as the moderator indicated no significant overall main effects of physical neglect (β = 0.010, S.E. = 0.006, t (1, 89) = 1.62, *p* = 0.110), sex assigned at birth (β = 0.007, S.E. = 0.004, t (1, 89) = 1.55, *p* = 0.126), or stress condition (β = 0.006, S.E. = 0.005, t (1, 89) = 1.39, *p* = 0.168); see overall marginal effects in the bottom right part of Table 5. However, we again found a significant three-way interaction of stress condition by sex assigned at birth by physical neglect history on peak BAC during ad libitum drinking (see right side of Table 3), β = −0.063, S.E. = 0.023, t (1, 89) = −2.70, *p* < 0.009 **. This three-way interaction is shown in Figure 2A,B. To probe the significant three-way interactions, we again examined simple effects tests comparing mean levels versus mean +1 unit greater physical neglect, highlighting sex-assigned-at-birth differences concerning peak BACs reached during the 90 min ad libitum drinking period.

*Simple effects for physical neglect (see right side of Table 5):* Only one simple main effect of physical neglect was significant for peak BAC. For men in the no stress condition there was a significant simple effect of neglect level, β = 0.039, S.E. = 0.012, t (1, 89) = 3.18, *p* = 0.002 **; men at mean +1 unit greater levels of physical neglect had (0.094) peak BAC compared to those at mean levels (0.055).

*Simple effects for physical neglect (see right side of Table 5):* There was also a single significant simple main effect of the stress condition. For men with +1 unit greater physical neglect scores, the stress condition was associated with lower peak BAC (0.050) versus (0.094) for those in the no stress condition; B = −0.044, S.E. = 0.020, t (1, 89) = −2.16, *p* = 0.033 *.

*Simple effects for sex differences (see right side of Table 5):* Men in the no stress condition at mean +1 unit greater physical neglect reached higher peak BACs (0.094) than did women (0.038); β = 0.056, S.E. = 0.018, t (1, 89) = 3.19, *p* = 0.002 **. Thus, men in the no stress condition with the higher levels of physical neglect reached the beyond-legal-drinking limit with BAC levels at 0.094; this is risky heavy episodic drinking reflecting “*state*” behavioral IC. The remaining simple main effects of sex were not significant.

### 3.3. Models: Life Events as a Predictor and Physical Neglect as a Covariate

#### 3.3.1. Model: Life Events as a Predictor and Physical Neglect as a Covariate for Drink Orders

Parameter estimates for tested effects concerning life events as a predictor and physical neglect as a covariate for the number of standardized drink orders can be found on the left side of Table 4. None of the hypothesized interactions were significant. This suggests that life events do not moderate how acute stress and sex assigned at birth associate with standardized drink orders. In this model, where physical neglect was entered as a covariate, the overall association of physical neglect and drink orders accounting for other main and interaction effects was significant: β = 0.491, S.E. = 0.235; t (1, 90) = 2.09, *p* = 0.0039 *. As with the models for drink orders with physical neglect as the moderator, we found a significant overall main effect of sex assigned at birth in the model using life events as the moderator. As in the earlier model, men ordered about 0.8 more standardized drinks [(2.6 drinks); β = 0.736, S.E. 0.177, t (1, 89) = 4.16, *p* < 0.001 ***] than did women (1.8 drinks). In contrast to the prior models, the model with life events as moderator indicated a significant overall main effect of stress: β = 0.446, S.E. 0.207, t (1, 89) = 2.15, *p* = 0.034 *. Participants in the stress condition ordered an average of 2.4 drinks compared to 2.0 for those in the no stress condition. The overall effect of life events was not significant (β = −0.025, S.E. 0.027, t (1, 89) = −0.91, ns).

#### 3.3.2. Model: Life Events as a Predictor and Physical Neglect as a Covariate for Peak BAC

Parameter estimates for tested effects concerning life events as a predictor and physical neglect as a covariate for peak BAC during the 90-minute ad libitum drinking session can be found on the right side of Table 4. None of the hypothesized interactions were significant. This suggests that the associations of peak BAC with stress and sex assigned at birth are not conditional on the life events measures. None of the overall main effects of stress, sex, or life events were significant in the model, including all other main and interaction effects (sex: β = 0.006, S.E. = 0.004, t (1, 89) = 1.43, *p* = 0.157; stress: β = 0.008, S.E. = 0.005, t (1, 89) = 1.69, *p* = 0.095, life events:β = −0.000, S.E. = 0.001, t (1, 89) = −0.61, *p* = 0.540).

## 4. Discussion

Our experiment addressed women’s health disparities by comparing two theoretical frameworks of AUD: (1) Self-Medication and (2) Behavioral Economic Models. Our study supported the Self-Medication Model with evidence that women with a history of physical neglect exhibit behavioral IC over alcohol under an acute stress manipulation, yet there was no clear evidence of this for men. Our findings are somewhat consistent with Chaplin et al. (2018) who proposed that chronic life stress in childhood would lead to increased acute stress reactivity among women yet reduce reactivity among men. We build upon Chaplin et al.’s (2018) ideas concerning the Self-Medication Model by illustrating the role of physical neglect as a particularly important type of chronic stress that exaggerates the response to acute stress among women. Although we set out to find out new information regarding women, we ended up discovering something new and important regarding physically neglected men. Surprisingly, our findings supported the Behavioral Economics Model with evidence that physically neglected men exhibited behavioral IC in the absence of an acute stress manipulation. Our study suggests that men who experienced more physical neglect prior to the age of 12 may be uniquely vulnerable to AUD despite other contextual factors (e.g., acute stress). We also found that physical neglect was more predictive of behavioral IC than other life event histories (i.e., 46-items), signifying that this short physical neglect measure (i.e., 5-items) may be an efficient direction for exploring chronic stress in future research. This study proposes that more competing theory tests ought to be conducted within ad libitum methodologies to move both theory and therapies forward.

### 4.1. The Self-Medication Model

Conceivably, the inability to resist drinking after experiencing stress and/or negative emotions may reflect a broader self-medication process underlying excessive alcohol use. Consistent with the Self-Medication Model, our findings suggest that acute stress increases alcohol consumption among women. Specifically, women in the acute stress (TSST) condition ordered more standardized drinks and achieved higher peak BACs than those in the no stress (no TSST) condition. This pattern aligns with prior work showing that women may be more likely than men to consume alcohol in the face of uncomfortable emotions (McCaul et al., 2019). More broadly, stress and negative emotions are robustly associated with drinking initiation, maintenance, and relapse among women (Koob & White, 2017; Peltier et al., 2019).

Our findings concerning our two distinct measures of chronic stress history were mixed. We found no evidence in favor of our original hypothesis that those who have experienced more negative life events would exhibit more behavioral IC than those who have experienced fewer negative life events (Guidi et al., 2020; Widom et al., 2015); these cited studies used the Life Events Checklist (McEwen & Stellar, 1993) rather than the Life Experiences Survey (Johnson & McCutcheon, 1980) that we used in our study. One possible explanation for these mixed findings is differences in measures across studies. Furthermore, our findings are inconsistent with Zhao et al. (2023) who found negative life events (measured with the Life Experiences Survey) were linked to increased alcohol use. In addition, our measure of chronic negative life events history was not found to be a significant moderator concerning the TSST task and/or sex assigned at birth on our ad libitum drinking session outcome variables (i.e., standardized drink orders and peak BAC). Nevertheless, our findings are highly consistent with Portillo et al. (2023), who found no mean differences among 48 males with an AUD versus 17 men with no history of any substance use disorders using the cumulative stress index.

Our second measure of chronic stress was physical neglect history before the age of twelve, which played a much more prominent role in our findings. Thus, our findings regarding the importance of physical neglect in AUD formation are consistent with Rubio et al. (2025). Likewise, our findings are highly consistent with cross-sectional studies showing a link between physical neglect and perceived IC (Bitsoih et al., 2023; Noudali et al., 2022; Patock-Peckham et al., 2020). In particular our study was highly consistent with Patock-Peckham et al.’s (2020) study which showed the link between physical neglect and perceived IC was slightly stronger for cisgender men than for women. This current study is the first to show the importance of physical neglect and behavioral IC during an ad libitum drinking session.

While the Self-Medication Model does have a valuable place in the study of the etiology of AUDs, it neglects to explain all pathways to behavioral IC. Specifically, the model does not explain why physically neglected men in the no stress condition exhibited behavioral IC but reduced their drinking while in the stress condition. Our findings are inconsistent with the Self-Medication Model such that higher physical neglect histories alone without acute stress robustly influenced men to drink beyond recommended limits. Thus, the Self-Medication Model cannot account for our findings concerning physically neglected cisgender men in our sample.

### 4.2. The Behavioral Economics Model

Our findings are consistent with the Behavioral Economics Model but not how we expected. In our experiment, men in the no stress (no TSST) group with higher physical neglect histories reached beyond the 0.080 BAC legal limit at 0.094 peak BAC and ordered an additional average of 3.36 standard drinks during the 90 min ad libitum (free) drinking event regardless of whether they received the 0.065 BAC alcohol prime (three standardized cocktails) or an expectancy beverage 0.000 BAC (three mocktails) prior to the drinking session. Higher physical neglect history scores while controlling for life events in the no stress (no TSST) condition led men to exhibit behavioral IC during the drinking session. Women who were in this same category [higher physical neglect scores in the no stress (no TSST) condition] ordered on average only 1.77 standard drinks and achieved an average peak BAC of 0.038 during the ad libitum drinking session regardless of their prime condition. These findings suggest that the Behavioral Economics Model is more relevant to men overall under this context and yet fails to explain the choices of physically neglected women who were not administered the stress task. Thus, how physical neglect contributes to AUD development in emerging adulthood should be given more research attention than it has received; we must consider sex differences.

Current conceptualizations of Behavioral Economics applied to alcohol do not make unique or distinct predictions based upon sex assigned at birth. This is due in part to enormous health disparities in alcohol self-administration studies which over-represented men and grossly under-represented women even when studying stress manipulations (Patock-Peckham et al., 2022, see p. 3; Weckesser et al., 2025). Classic alcohol self-administration studies that we all base our theoretical stances on only studied men or too few women to even analyze as a variable. Our results suggest that sex assigned at birth must be considered in such explorations of dysregulated drinking to capture the true etiology of “*state*” behavioral IC within a social context.

Our findings suggest that the Behavioral Economic Model may be of particular importance in the study of AUD risk among men. Our experiment extends this theory by demonstrating that physical neglect prior to the age of 12 moderated “*state*” behavioral IC in emerging adult men, despite clear incentives to limit alcohol consumption to no more than one standard drink per hour. Prior self-administration studies have shown that participants drink more moderately in group contexts when monetary incentives for limiting alcohol consumption are introduced (Leeman et al., 2013). However, this pattern did not appear to hold for men with higher levels of physical neglect, suggesting that early trauma in the form of physical neglect may diminish the effectiveness of external behavioral constraints on drinking behavior. Our findings suggest that intervention and prevention efforts should target physically neglected men as a potential way to combat the development of AUD. Future research should examine the mechanisms underlying this association to better identify opportunities for disrupting pathways from early neglect to behavioral IC and AUD risk among physically neglected men. Notably, our findings concerning women were less consistent with the Behavioral Economic Model. Specifically, physically neglected women appeared to require acute stress exposure to exhibit behavioral IC, whereas physically neglected men demonstrated behavioral IC even in the absence of acute stress. This pattern suggests that the existing Behavioral Economics Model may not fully account for sex-specific pathways linking childhood adversity to alcohol misuse and underscores the important of including adequately powered samples of both men and women in future alcohol self-administration studies.

### 4.3. Limitations of the Current Study

There are several limitations to the present study. First, future studies should strive to specifically recruit and oversample individuals with very extreme physical neglect histories from an older community sample diagnosed with AUD to replicate our findings. Second, we measured childhood physical neglect retrospectively from our adult participants. Despite evidence suggesting that retrospective reports of childhood trauma, including physical neglect, are reliable (Pinto et al., 2014), future studies should follow participants from childhood to young adulthood to corroborate our findings reflecting the influence of childhood physical neglect on stress and drinking. Third, while our sample did include individuals from the community at large, a substantial number of our participants did attend our home university. Presumably, physical neglect trauma histories would have been more severe in a less educated sample on average (Maguire-Jack & Font, 2017; Vandermiden et al., 2019). Future research should also examine more racially diverse samples than this one so that generalizability is less restricted. Fourth, we were required to screen for mental health disorders such as depression, suicidal ideation, and generalized anxiety symptoms due to our IRB requirements at a university void of a department of psychiatry. Thus, future studies should seek to include samples of individuals with greater levels of depression and generalized anxiety symptoms, especially if this work can be performed with appropriate psychiatric staff on call. Fifth, future research teams are strongly encouraged to build on this work by using event-level methodologies (e.g., EMA) to better approximate behavior in real-life social settings and account for greater levels of drinking. Lastly, we must acknowledge that the Self-Medication and Behavioral Economics Models are not mutually exclusive. Yet these theories when used together in the same study can elucidate where new theories encapsulating sex difference should go next.

Despite the aforementioned limitations, this study includes some clear methodological advantages. For instance, emerging adults in social settings consume approximately 60% of all alcohol purchased (Creswell et al., 2012; Fairbairn & Sayette, 2014; Sayette et al., 2016). Yet, most (~90%) ad libitum drinking session studies were conducted with solitary drinking paradigms (Clay & Parker, 2018; Creswell & Fairbairn, 2025; Cyders et al., 2016; Higgins & Marlatt, 1973; Hull & Young, 1983; McGrath et al., 2016; Miller et al., 1974; Pratt & Davidson, 2009; Thomas et al., 2011). Despite enormous societal costs associated with alcohol use (i.e., $1306 per adult globally; Manthey et al., 2021), competing theory tests in well-powered studies of observed heavy episodic behaviors while in a social drinking context are rare (Patock-Peckham et al., 2022; see p. 3). Thus, there are several strategic advantages to this current investigation relative to similar studies. First, we conducted our study in a social drinking context which better reflects most drinking behaviors in emerging adulthood, compared to the utilization of much more convenient solitary drinking contexts or retrospective surveys. Second, our sample size was sufficiently powered, to study sex assigned at birth as a possible interacting (moderating) variable. We were able to detect differences due to sex assigned at birth while employing multilevel modeling to account for both individual and group-level dynamics. To our knowledge, this is the first study to investigate the interactions among acute stress (i.e., TSST), two types of distinct chronic stress (e.g., physical neglect histories and negative life events), as well as sex assigned at birth in a realistic social drinking context. Third, we provided monetary incentives for individuals to limit their drinking during the 90 min ad libitum free drinking session to model behavioral IC. Fourth, we measured in full standard drinks which is an easier unit for laypeople to understand rather than the difficult-to-understand millimeter measurements utilized in most two-drink taste test experiments. Fifth, we did allow our participants to reach a true heavy episodic drinking cut-off level (i.e., BAC up to 0.120) to fully measure “*state*” behavioral IC in a controlled, safe experimental environment. We believe that allowing for a higher BAC limit of 0.120 (i.e., 6+ standardized drinks) in a short 90 min timeframe enhanced the ecological validity of our study to understand risky drinking behaviors in a realistic social drinking context.

### 4.4. Conclusions

In conclusion, acute stress may exacerbate “*state*” behavioral IC among physically neglected women, whereas physically neglected men exhibit behavioral IC in the absence of acute stress. According to Cicchetti and Handley (2019), clarifying the processes underlying the developmental etiology of AUD among children exposed to early adversity is critical for not only informing developmental theories but for designing effective prevention and intervention programs. While we agree with Cicchetti and Handley (2019), we also suggest that it is important to do critical tests that pit existing theoretical stances directly against one another (see Kuhn, 2012) within social drinking contexts with samples well-powered to fully examine key demographics, like sex assigned at birth and beyond. Taking on such a resource-heavy strategy is likely to challenge even the most well-vetted existing theories of AUDs. This will hopefully lead to newer more comprehensive theories that account for sex differences such as those found in this study. To conduct Kuhn (2012)-paradigm shifting research generating new theoretical perspectives that could potentially advance the science around AUD faster than it has moved in the past 50 years, we must use sample sizes large enough to detect important interactions with key variables such as sex assigned at birth and trauma history.

The Self-Medication Model did not explain why acute stress reduces drinking among men but accelerates it among women. Moreover, the Behavioral Economics Model of AUD did not explain why only physically neglected men but not women in the no stress condition were likely to exhibit behavioral IC beyond legal limits despite clear incentives to limit alcohol consumption. Neither theory fully accounts for our findings; thus, we call for revision of these major theories with the replication of these findings here. Our classic theoretical stances on AUD have been grossly hampered by a lack of women in oral alcohol self-administration studies. It is time to make health disparity research concerning behavioral IC a priority.

## Figures and Tables

**Table 1:** Sociodemographic data, average drinking habits, and average scores of key variables.

Variable	Stress Condition (*n* = 108)	No Stress Condition (*n* = 102)	Overall (*n* = 210)	*p*-Value
Percent male	49.1%	51.0%	50.0%	0.78
Race				0.97
White	70.4%	71.6%	71.6%	
African American	7.4%	8.8%	8.1%	
Native American/Alaskan Native	3.7%	4.9%	4.3%	
Asian	12.0%	9.8%	11.0%	
Native Hawaiian or Pacific Islander	1.9%	1.0%	1.4%	
Other	3.7%	2.9%	3.3%	
Ethnicity				0.58
Hispanic	23.1%	26.5%	24.8%	
Non-Hispanic	76.9%	73.5%	75.2%	
Student status				0.70
Full-time	78.7%	76.5%	77.6%	
Non-student	21.2%	23.5%	22.4%	
Past 30-day alcohol frequency/quantity reported at screening: TLFB; M (SD)				
Frequency of any use	6.83 (4.77)	6.80 (5.75)	6.82 (5.25)	0.97
Frequency of heavy use	2.49 (2.38)	2.61 (2.70)	2.55 (2.54)	0.74
Drinks per drinking day	3.90 (1.80)	4.15 (2.32)	4.02 (2.07)	0.38
Total number of drinks	26.19 (21.83)	26.86 (23.68)	26.51 (22.69)	0.83
Covariates				
Usual drinking quantity	2.58 (0.80)	2.78 (0.92)	2.69 (0.86)	0.10
Anxiety sensitivity	1.98 (0.54)	2.15 (0.64)	2.06 (0.60)	0.04 *
Moderators				
Physical neglect	1.20 (0.37)	1.22 (0.42)	1.21 (0.39)	0.77
Life events history	6.97 (3.19)	7.03 (3.75)	7.00 (3.45)	0.90

Note—those randomly assigned to the no stress/no TSST condition had significantly higher anxiety sensitivity than those randomly assigned to the stress/TSST condition prior to our administration of the Trier Social Stress Test, F(1, 208) = 4.27, *p* = 0.04 *. Anxiety sensitivity was included as a covariate so, even if there was a difference across the stress conditions this was covaried out. All other variables measured prior to the ad libitum drinking lab day yielded non-significant differences.

**Table 2:** Summary of Impaired-Control-Over-Alcohol (IC) Assessments: Survey versus Behavioral IC.

**Survey or Trait IC**	
ICS—Part I	(5 items; Heather et al., 1993, p. 704)
Attempted	Self-report survey that assesses attempts to control alcohol use in the past 6 months.
Control	*Example item:* “During the last six months, I have tried to stop drinking for a period of time”.
ICS—Part II	(10 items; Heather et al., 1993, p. 704)
Failed	Self-report survey that assesses the degree of success in controlling drinking in the past 6 months.
	*Example item:* “During the last six months, I have started drinking even after deciding not to.”
ICS—Part III	(10 items; Heather et al., 1993, p. 704)
Perceived Control	Self-report survey that assesses beliefs about one’s perceived future control over drinking. *Example item:* “Even if I intended to have only one or two drinks, I would end up having many more.”
**State IC**	
Behavioral IC (Leeman et al., 2013; Patock-Peckham et al., 2022)	
	Direct observations of behavior or individual(s) drinking beyond recommended and/or instructed limits for drinking during a specific period with clear incentives to refrain from overconsuming alcohol during an ad libitum (free drinking session in a bar setting). We define behavioral IC as choosing the immediate reward of another drink right now versus the uncertain long-term reward of a better subject payment at the end of the drinking event. As such behavioral IC is a representation of a form of delayed discounting with alcohol as the more immediate reward and the extra money at the end of the night as the uncertain long-term (several hours away) reward.

*Note*. ICS = Impaired Control Scale.

**Table 3:** Type III Fixed Effects for Selected Effects Concerning the Physical Neglect Model with Life Events as a Covariate.

	Number of Standard Drinks Ordered During the 90 min Ad Libitum Drinking Session					Peak BAC				
**Main Effects**	**β**	**S.E.**	**df**	**t Value**	***p* Value**	**β**	**S.E.**	**df**	**t Value**	***p* Value**
Trier Social Stress Test	0.437	0.270	t (1, 90)	=1.62	*p* = 0.109	0.010	0.006	t (1, 90)	=1.69	*p* = 0.094
Cisgender Sex	**0.807**	**0.252**	**t (1, 90)**	**=3.20**	*p* = 0.002 **	0.011	0.006	t (1, 89)	=1.78	*p* = 0.079
Physical Neglect	0.018	0.427	t (1, 90)	=0.40	*p* = 0.969	−0.006	0.010	t (1, 89)	=−0.60	*p* = 0.547
**Interactions**										
Trier by Sex	−0.128	0.346	t (1, 90)	=−0.37	*p* = 0.712	−0.008	0.008	t (1, 89)	=−1.01	*p* = 0.316
Trier by Physical Neglect	0.934	0.560	t (1, 90)	=1.67	*p* = 0.099	0.017	0.014	t (1, 89)	=1.24	*p* = 0.219
Sex by Physical Neglect	0.788	0.659	t (1, 90)	=1.20	*p* = 0.236	**0.045**	**0.016**	**t (1, 89)**	**=2.82**	*p* = 0.006 **
Trier by Sex by Phys Neg	**−2.05**	**0.955**	**t (1, 90)**	**=−2.14**	*p* = 0.035 *	**−0.063**	**0.023**	**t (1, 89)**	**=−2.70**	*p* = 0.009 **
**Covariates**										
Alcohol	−0.256	0.218	t (1, 90)	=−1.21	*p* = 0.230	**0.028**	**0.017**	**t (1, 90)**	**=−1.66**	*p* = 0.010 **
Life Events	0.003	0.025	t (1, 90)	=0.10	*p* = 0.921	0.000	0.001	t (1, 89)	=0.16	*p* = 0.872
Anxiety Sensitivity	0.201	0.142	t (1, 90)	=1.42	*p* = 0.159	0.006	0.003	t (1, 89)	=1.88	*p* = 0.064
Usual Drinking Quantity	0.170	0.103	t (1, 90)	=1.65	*p* = 0.103	0.004	0.003	t (1, 89)	=1.47	*p* = 0.144
BAC at the start of ad lib						0.181	0.247	t (1, 89)	=0.73	*p* = 0.466

*Note.* * = *p* <0.05; ** = *p* < 0.01.

**Table 4:** Type III Fixed Effects for Selected Effects Concerning Life Events with Physical Neglect as a Covariate.

	Number of Standard Drinks Ordered During the 90 min Ad Libitum Drinking Session					Peak BAC				
**Main Effects**	**β**	**S.E.**	**df**	**t Value**	***p* Value**	**β**	**S.E.**	**df**	**t Value**	***p* Value**
Trier Social Stress Test	0.481	0.269	t (1, 90)	=1.79	*p* = 0.077	0.011	0.006	t (1, 90)	=1.71	*p* = 0.091
Cisgender Sex	**0.787**	**0.255**	**t (1, 90)**	**=3.09**	***p* = 0.003** **	0.092	0.006	t (1, 89)	=1.47	*p* = 0.144
Life Events	0.031	0.041	t (1, 90)	=0.76	*p* = 0.450	0.004	0.001	t (1, 89)	=0.41	*p* = 0.681
**Interactions**										
Trier by Sex	−0.080	0.348	t (1,90)	=−0.23	*p* = 0.818	−0.006	0.009	t (1, 89)	=−0.70	*p* = 0.485
Trier by Life Events	−0.010	0.059	t (1, 90)	=−0.16	*p* = 0.872	−0.000	0.001	t (1, 89)	=−0.10	*p* = 0.922
Sex by Life Events	−0.074	0.072	t (1, 90)	=−1.02	*p* = 0.312	−0.002	0.002	t (1, 89)	=−0.88	*p* = 0.383
Trier by Sex by Life Events	−0.062	0.106	t (1, 90)	=−0.59	*p* = 0.560	0.000	0.003	t (1, 89)	=0.05	*p* = 0.960
**Covariates**										
Alcohol	−0.277	0.210	t (1, 90)	=−1.32	*p* = 0.191	0.025	0.017	t (1, 90)	=1.46	*p* = 0.148
Physical Neglect	**0.491**	**0.235**	**t (1, 90)**	**=2.09**	***p* = 0.039** *	0.009	0.006	t (1, 89)	=1.57	*p* = 0.119
Anxiety Sensitivity	0.201	0.145	t (1, 90)	=1.39	*p* = 0.168	0.006	0.004	t (1, 89)	=1.77	*p* = 0.080
Usual Drinking Quantity	0.183	0.104	t (1, 90)	=1.76	*p* = 0.081	0.004	0.003	t (1, 89)	=1.61	*p* = 0.112
BAC at the start of ad lib						0.220	0.252	t (1, 89)	=0.87	*p* = 0.386

*Note.* * = *p* < 0.05; ** = *p* < 0.01.

**Table 5:** Model for physical neglect with life events as a covariate: simple 2-way interactions, simple main effects, point estimates, and overall marginal effects.

	Drink Orders					Peak BAC				
**Label**	**Estimate**	**S.E.**	**df**	**t Value**	***p* Value**	**Estimate**	**S.E.**	**df**	**t Value**	***p* Value**
**Simple 2-way interactions**										
Stress by neglect, women	0.934	0.560	1, 90	1.67	0.099	0.017	0.014	1, 89	1.24	0.219
Stress by neglect, men	−1.112	0.757	1, 90	−1.47	0.145	−0.046	0.019	1, 89	−2.49	**0.015** *
**Simple Main Effects**										
Neglect at women, no stress	0.017	0.427	1, 90	0.04	0.969	−0.006	0.010	1, 89	−0.60	0.547
Neglect at men, no stress	0.805	0.501	1, 90	1.61	0.112	0.039	0.012	1, 89	3.18	0.002 **
Neglect at women, stress	0.950	0.376	1, 90	2.53	0.013 *	0.010	0.009	1, 89	1.17	0.246
Neglect at men, stress	−0.308	0.574	1, 90	−0.54	0.593	−0.007	0.014	1, 89	−0.50	0.615
Stress at women, mean neglect	0.437	0.270	1, 90	1.62	0.109	0.010	0.006	1, 89	1.69	0.094
Stress at men, mean neglect	0.309	0.272	1, 90	1.14	0.259	0.002	0.006	1, 89	0.32	0.748
Stress at women, +1 unit neglect	1.370	0.612	1, 90	2.24	0.028 *	0.027	0.015	1, 89	1.85	0.068
Stress at men, +1 unit neglect	−0.803	0.837	1, 90	−0.96	0.340	−0.044	0.020	1, 89	−2.16	0.033 *
Sex at no stress, mean neglect	0.807	0.252	1, 90	3.20	0.002 **	0.011	0.006	1, 89	1.78	0.079
Sex at stress, mean neglect	0.679	0.238	1, 90	2.85	0.005 **	0.002	0.006	1, 89	0.39	0.696
Sex at no stress, +1 unit neglect	1.595	0.722	1, 90	2.21	0.030 *	0.056	0.018	1, 89	3.19	0.002 **
Sex at stress, +1 unit neglect	−0.578	0.743	1, 90	−0.78	0.438	−0.015	0.018	1, 89	−0.84	0.403
**Point Estimates**										
Women, mean neglect, no stress	1.752	0.226	1, 90	7.74	<0.001 ***	0.044	0.005	1, 89	8.63	<0.001 ***
Women, +1 unit neglect, no stress	1.769	0.485	1, 90	3.65	<0.001 ***	0.038	0.012	1, 89	3.24	<0.002 **
Men, mean neglect, no stress	2.559	0.223	1, 90	11.45	<0.001 ***	0.055	0.005	1, 89	10.89	<0.001 ***
Men, +1 unit, no stress	3.364	0.561	1, 90	5.99	<0.001 ***	0.095	0.014	1, 89	6.92	<0.001 ***
Women, mean neglect, stress	2.189	0.210	1, 90	10.42	<0.001 ***	0.055	0.005	1, 89	11.45	<0.001 ***
Women, +1 unit, stress	3.139	0.424	1, 90	7.40	<0.001 ***	0.065	0.010	1, 89	6.46	<0.001 ***
Men, mean neglect, stress	2.868	0.218	1, 90	13.16	<0.001 ***	0.057	0.005	1, 89	11.46	<0.001 ***
Men, +1 unit neglect, stress	2.561	0.636	1. 90	4.02	<0.001 ***	0.050	0.015	1, 89	3.22	<0.002 **
**Overall (Marginal) Effects**										
Physical neglect centered	0.377	0.243	1, 90	1.55	0.124	0.010	0.006	1, 89	1.62	0.110
Sex assigned at birth	0.743	0.174	1, 90	4.27	**<0.001** ***	0.007	0.004	1, 89	1.55	0.126
Acute stress (Trier, no Trier)	0.375	0.209	1, 90	1.80	0.076	0.006	0.005	1, 89	1.39	0.168

*Note*. * *p* < 0.05; ** *p* < 0.01; *** *p* < 0.001.

## Data Availability

Data available upon request of the corresponding author.

## References

[B1-behavsci-16-01342] Acuff S. F., Tucker J. A., Murphy J. G. (2021). Behavioral economics of substance use: Understanding and reducing harmful use during the COVID-19 Pandemic. Experimental and Clinical Psychopharmacology.

[B2-behavsci-16-01342] Allison P. D. (1990). Change scores as dependent variables in regression analysis. Sociological Methodology.

[B3-behavsci-16-01342] Alvarez-Alonso M. J., Jurado-Barba R., Martinez-Martin N., Espin-Jaime J. C., Bolaños-Porrero C., Ordoñez-Franco A., Rodriguez-Lopez J. A., Lora-Pablos D., de la Cruz-Bértolo J., Jimenez-Arriero M. A., Manzanares J., Rubio G. (2016). Association between maltreatment and polydrug use among adolescents. Child Abuse & Neglect.

[B4-behavsci-16-01342] Amlung M., MacKillop J. (2014). Understanding the effects of stress and alcohol dues on motivation for alcohol via behavioral economics. Alcoholism, Clinical and Experimental Research.

[B5-behavsci-16-01342] Bellis M. D. D. (2001). Developmental traumatology: The psychobiological development of maltreated children and its implications for research, treatment, and policy. Development and Psychopathology.

[B6-behavsci-16-01342] Bernstein D. P., Fink L., Handelsman L., Foote J., Lovejoy M., Wenzel K., Sapareto E., Ruggiero J. (1994). Initial reliability and validity of a new retrospective measure of child abuse and neglect. The American Journal of Psychiatry.

[B7-behavsci-16-01342] Bernstein D. P., Stein J. A., Newcomb M. D., Walker E., Pogge D., Ahluvalia T., Stokes J., Handelsman L., Medrano M., Desmond D., Zule W. (2003). Development and validation of a brief screening version of the Childhood Trauma Questionnaire. Child Abuse & Neglect.

[B8-behavsci-16-01342] Bickel W. K., Jarmolowicz D. P., MacKillop J., Epstein L. H., Carr K., Mueller E. T., Waltz T. J. (2012). The behavioral economics of reinforcement pathologies: Novel approaches to addictive disorders. APA addiction syndrome handbook, Vol. 2: Recovery, prevention, and other issues.

[B9-behavsci-16-01342] Bickel W. K., Marsch L. A. (2001). Toward a behavioral economic understanding of drug dependence: Delay discounting processes. Addiction.

[B10-behavsci-16-01342] Bickel W. K., Stein J. S., Moody L. N., Snider S. E., Mellis A. M., Quisenberry A. J., Stevens J. R. (2017). Toward narrative theory: Interventions for reinforcer pathology in health behavior. Impulsivity: How time and risk influence decision making.

[B11-behavsci-16-01342] Bitsoih J., Patock-Peckham J. A., Canning J. R., Ong A., Becerra A., Broussard M. (2023). Do coping motives and perceived impaired control mediate the indirect links from childhood trauma facets to alcohol-related problems?. Behavioral Sciences.

[B12-behavsci-16-01342] CDC (2021). Alcohol and public health: Alcohol-related disease impact. Annual average for United States 2020–2021 alcohol-attributable deaths due to excessive alcohol use, all ages.

[B13-behavsci-16-01342] Chaplin T. M., Niehaus C., Gonçalves S. F. (2018). Stress reactivity and the developmental psychopathology of adolescent substance use. Neurobiology of Stress.

[B14-behavsci-16-01342] Chung T., Martin C. S. (2002). Concurrent and discriminant validity of DSM-IV symptoms of impaired control over alcohol consumption in adolescents. Alcoholism: Clinical and Experimental Research.

[B15-behavsci-16-01342] Cicchetti D., Handley E. D. (2019). Child maltreatment and the development of substance use and disorder. Neurobiology of Stress.

[B16-behavsci-16-01342] Clay J. M., Parker M. O. (2018). The role of stress-reactivity, stress-recovery and risky decision-making in psychosocial stress-induced alcohol consumption in social drinkers. Psychopharmacology.

[B17-behavsci-16-01342] Cohen J. R., Menon S. V., Shorey R. C., Le V. D., Temple J. R. (2017). The distal consequences of physical and emotional neglect in emerging adults: A person-centered, multi-wave, longitudinal study. Child Abuse & Neglect.

[B18-behavsci-16-01342] Conger J. J. (1956). Reinforcement theory and the dynamics of alcoholism. Quarterly Journal of Studies on Alcohol.

[B19-behavsci-16-01342] Creswell K. G. (2021). Drinking together and drinking alone: A social-contextual framework for examining risk for alcohol use disorder. Current Directions in Psychological Science.

[B20-behavsci-16-01342] Creswell K. G., Chung T., Clark D. B., Martin C. S. (2014). Solitary alcohol use in teens is associated with drinking in response to negative affect and predicts alcohol problems in young adulthood. Clinical Psychological Science.

[B21-behavsci-16-01342] Creswell K. G., Fairbairn C. E. (2025). The need for multi-participant alcohol administration studies. Addiction.

[B22-behavsci-16-01342] Creswell K. G., Sayette M. A., Manuck S. B., Ferrell R. E., Hill S. Y., Dimoff J. D. (2012). Drd4 polymorphism moderates the effect of alcohol consumption on social bonding. PLoS ONE.

[B23-behavsci-16-01342] Cullins E. C., Gunawan T., Schwandt M. L., Luk J. W., George D. T., Diazgranados N., Goldman D., Ramchandani V. A. (2025). Markers of negative emotionality in individuals With comorbid alcohol use disorder and post-traumatic stress disorder: Role of childhood trauma. Addiction Biology.

[B24-behavsci-16-01342] Curtin J. J., Fairchild B. A. (2003). Alcohol and cognitive control: Implications for regulation of behavior during response conflict. Journal of Abnormal Psychology.

[B25-behavsci-16-01342] Cyders M. A., Smith G. T., Spillane N. S., Fischer S., Annus A. M., Peterson C. (2007). Integration of impulsivity and positive mood to predict risky behavior: Development and validation of a measure of positive urgency. Psychological Assessment.

[B26-behavsci-16-01342] Cyders M. A., VanderVeen J. D., Plawecki M., Millward J. B., Hays J., Kareken D. A., O’Connor S. (2016). Gender specific effects of mood on alcohol seeking behaviors: Preliminary findings using intravenous alcohol self-administration. Alcoholism: Clinical and Experimental Research.

[B27-behavsci-16-01342] Davis J. P., Pedersen E. R., Tucker J., Dunbar M., Rodriguez A., Seelam R., D’Amico E. J. (2023). Childhood adversity and developmental trajectories of alcohol and cannabis co-use. Child Abuse & Neglect.

[B28-behavsci-16-01342] de Wit H., Söderpalm A. H. V., Nikolayev L., Young E. (2003). Effects of acute social stress on alcohol consumption in healthy subjects. Alcoholism: Clinical and Experimental Research.

[B29-behavsci-16-01342] Dong C., Wang Z., Jia F., Tian H., Zhang Y., Liu H., Yu X., Wang L., Fu Y. (2024). Gender differences in the association between childhood maltreatment and the onset of major depressive disorder. Journal of Affective Disorders.

[B30-behavsci-16-01342] Dube S. R., Miller J. W., Brown D. W., Giles W. H., Felitti V. J., Dong M., Anda R. F. (2006). Adverse childhood experiences and the association with ever using alcohol and initiating alcohol use during adolescence. Journal of Adolescent Health.

[B31-behavsci-16-01342] Ebbert A. M., Patock-Peckham J. A., Luk J. W., Voorhies K., Warner O., Leeman R. F. (2018). The mediating role of anxiety sensitivity in uncontrolled drinking: A look at gender-specific parental influences. Alcoholism: Clinical and Experimental Research.

[B32-behavsci-16-01342] Egry E. Y., Apostólico M. R., Albuquerque L. M., Gessner R., Fonseca R. M. G. S. d. (2015). Understanding child neglect in a gender context: A study performed in a Brazilian city. Revista Da Escola de Enfermagem Da USP.

[B33-behavsci-16-01342] Fairbairn C. E., Sayette M. A. (2014). A social-attributional analysis of alcohol response. Psychological Bulletin.

[B34-behavsci-16-01342] Finkelhor D., Turner H. A., Shattuck A., Hamby S. L. (2015). Prevalence of childhood exposure to violence, crime, and abuse: Results from the national survey of children’s exposure to violence. JAMA Pediatrics.

[B35-behavsci-16-01342] Gaume J., Murphy J. G., Studer J., Daeppen J.-B., Gmel G., Bertholet N. (2022). Behavioral economics indices predict alcohol use and consequences in young men at 4-year follow-up. Addiction.

[B36-behavsci-16-01342] GBD 2016 Alcohol Collaborators (2018). Alcohol use and burden for 195 countries and territories, 1990–2016: A systematic analysis for the Global Burden of Disease Study 2016. Lancet.

[B37-behavsci-16-01342] Goodman W. K., Janson J., Wolf J. M. (2017). Meta-analytical assessment of the effects of protocol variations on cortisol responses to the Trier Social Stress Test. Psychoneuroendocrinology.

[B38-behavsci-16-01342] Grant B. F., Dawson D. (2000). The alcohol use disorder and associated disabilities interview schedule-IV (AUDADIS-IV).

[B39-behavsci-16-01342] Guidi J., Lucente M., Sonino N., Fava G. A. (2020). Allostatic load and its impact on health: A systematic review. Psychotherapy and Psychosomatics.

[B40-behavsci-16-01342] Heather N., Tebbutt J. S., Mattick R. P., Zamir R. (1993). Development of a scale for measuring impaired control over alcohol consumption: A preliminary report. Journal of Studies on Alcohol.

[B41-behavsci-16-01342] Helminen E. C., Morton M. L., Wang Q., Felver J. C. (2021). Stress reactivity to the trier social stress test in traditional and virtual environments: A meta-analytic comparison. Psychosomatic Medicine.

[B42-behavsci-16-01342] Hersh M. A., Hussong A. M. (2009). The association between observed parental emotion socialization and adolescent self-medication. Journal of Abnormal Child Psychology.

[B43-behavsci-16-01342] Higgins R. L., Marlatt G. A. (1973). Effects of anxiety arousal on the consumption of alcohol by alcoholics and social drinkers. Journal of Consulting and Clinical Psychology.

[B44-behavsci-16-01342] Hox J., Balderjahn I., Mathar R., Schader M. (1998). Multilevel modeling: When and why. Classification, data analysis, and data highways.

[B45-behavsci-16-01342] Hull J. G., Young R. D. (1983). Self-consciousness, self-esteem, and success–failure as determinants of alcohol consumption in male social drinkers. Journal of Personality and Social Psychology.

[B46-behavsci-16-01342] Jernigan D. H., Trangenstein P. J. (2020). What’s next for WHO’s global strategy to reduce the harmful use of alcohol?. Bulletin of the World Health Organization.

[B47-behavsci-16-01342] Johnson J. H., McCutcheon S. M., Sarason I. G., Speilberger C. D. (1980). Assessing life stress in older children and adolescents: Preliminary findings with the Life Events Checklist. Stress and anxiety.

[B48-behavsci-16-01342] Kalina E., Boyd-Frenkel K., Patock-Peckham J. A., Schneidewent L., Broussard M. L., Leeman R. F. (2023). Does relationship-contingent self-esteem play a role in the stress to impaired control pathway to alcohol-related problems in a college student sample?. Behavioral Sciences.

[B49-behavsci-16-01342] Keyes K. M., Hatzenbuehler M. L., Hasin D. S. (2011). Stressful life experiences, alcohol consumption, and alcohol use disorders: The epidemiologic evidence for four main types of stressors. Psychopharmacology.

[B50-behavsci-16-01342] Keyes K. M., Martins S. S., Blanco C., Hasin D. S. (2010). Telescoping and gender differences in alcohol dependence: New evidence from two national surveys. American Journal of Psychiatry.

[B51-behavsci-16-01342] Khantzian E. J. (1997). The self-medication hypothesis of substance use disorders: A reconsideration and recent applications. Harvard Review of Psychiatry.

[B52-behavsci-16-01342] King K. M., Patock-Peckham J. A., Dager A. D., Thimm K., Gates J. R. (2014). On the mismeasurement of impulsivity: Trait, behavioral, and neural models in alcohol research among adolescents and young adults. Current Addiction Reports.

[B53-behavsci-16-01342] Kirschbaum C., Pirke K. M., Hellhammer D. H. (1993). The ‘Trier Social Stress Test’—A tool for investigating psychobiological stress responses in a laboratory setting. Neuropsychobiology.

[B54-behavsci-16-01342] Koob G. F., White A. (2017). Alcohol and the female brain. 2017 National Conference on Alcohol and Opioid Use in Women & Girls: Advances in Prevention, Treatment, and Recovery Research.

[B55-behavsci-16-01342] Kuhn T. S. (2012). The structure of scientific revolutions.

[B56-behavsci-16-01342] Leeman R. F., Beseler C. L., Helms C. M., Patock-Peckham J. A., Wakeling V. A., Kahler C. W. (2014). A brief, critical review of research on impaired control over alcohol use and suggestions for future studies. Alcoholism: Clinical and Experimental Research.

[B57-behavsci-16-01342] Leeman R. F., Corbin W. R., Nogueira C., Krishnan-Sarin S., Potenza M. N., O’Malley S. S. (2013). A human alcohol self-administration paradigm to model individual differences in impaired control over alcohol use. Experimental and Clinical Psychopharmacology.

[B58-behavsci-16-01342] Leeman R. F., Patock-Peckham J. A., Potenza M. N. (2012). Impaired control over alcohol use: An under-addressed risk factor for problem drinking in young adults?. Experimental and Clinical Psychopharmacology.

[B59-behavsci-16-01342] Leeman R. F., Toll B. A., Taylor L. A., Volpicelli J. R. (2009). Alcohol-induced disinhibition expectancies and impaired control as prospective predictors of problem drinking in undergraduates. Psychology of Addictive Behaviors.

[B60-behavsci-16-01342] Magrys S. A., Olmstead M. C. (2015). Acute stress increases voluntary consumption of alcohol in undergraduates. Alcohol and Alcoholism.

[B61-behavsci-16-01342] Maguire-Jack K., Font S. A. (2017). Community and individual risk factors for physical child abuse and child neglect: Variations by poverty status. Child Maltreatment.

[B62-behavsci-16-01342] Manthey J., Hassan S. A., Carr S., Kilian C., Kuitunen-Paul S., Rehm J. (2021). What are the economic costs to society attributable to alcohol use? A systematic review and modelling study. PharmacoEconomics.

[B63-behavsci-16-01342] Mazur J. E. (1993). Predicting the strength of a conditioned reinforcer: Effects of delay and uncertainty. Current Directions in Psychological Science.

[B64-behavsci-16-01342] McCaul M., Roach D., Hasin D., Weisner C., Chang G., Sinha R. (2019). Alcohol and women: A brief overview. Alcoholism: Clinical and Experimental Research.

[B65-behavsci-16-01342] McEwen B. S., Stellar E. (1993). Stress and the individual: Mechanisms leading to disease. Archives of Internal Medicine.

[B66-behavsci-16-01342] McGrath E., Jones A., Field M. (2016). Acute stress increases ad-libitum alcohol consumption in heavy drinkers, but not through impaired inhibitory control. Psychopharmacology.

[B67-behavsci-16-01342] Miller P. M., Hersen M., Eisler R. M., Hilsman G. (1974). Effects of social stress on operant drinking of alcoholics and social drinkers. Behaviour Research and Therapy.

[B68-behavsci-16-01342] Nagata J. M., Smith N., Sajjad O. M., Zamora G., Raney J. H., Ganson K. T., Testa A., Vittinghoff E., Jackson D. B. (2023). Adverse childhood experiences and sipping alcohol in U.S. Children: Findings from the Adolescent Brain Cognitive Development study. Preventive Medicine Reports.

[B69-behavsci-16-01342] National Institute on Alcohol Abuse and Alcoholism (2024). Alcohol consumption: Moderate and binge drinking.

[B70-behavsci-16-01342] Noudali S. N., Patock-Peckham J. A., Berberian S. L., Belton D. A., Campbell L. E., Infurna F. J. (2022). Does insomnia mediate the link between childhood trauma and impaired control over drinking, alcohol use, and related problems?. Addictive Behaviors Reports.

[B71-behavsci-16-01342] Oshri A., Kogan S. M., Kwon J. A., Wickrama K. a. S., Vanderbroek L., Palmer A. A., MacKillop J. (2018a). Impulsivity as a mechanism linking child abuse and neglect with substance use in adolescence and adulthood. Development and Psychopathology.

[B72-behavsci-16-01342] Oshri A., Liu S., Duprey E. B., MacKillop J. (2018b). Child maltreatment, delayed reward discounting, and alcohol and other drug use problems: The moderating role of heart rate variability. Alcoholism: Clinical and Experimental Research.

[B73-behavsci-16-01342] Patock-Peckham J. A., Belton D. A., D’Ardenne K., Tein J.-Y., Bauman D. C., Infurna F. J., Sanabria F., Curtis J., Morgan-Lopez A. A., McClure S. M. (2020). Dimensions of childhood trauma and their direct and indirect links to PTSD, impaired control over drinking, and alcohol-related-problems. Addictive Behaviors Reports.

[B74-behavsci-16-01342] Patock-Peckham J. A., Canning J. R., Leeman R. F. (2018). Shame is bad and guilt is good: An examination of the impaired control over drinking pathway to alcohol use and related problems. Personality and Individual Differences.

[B75-behavsci-16-01342] Patock-Peckham J. A., Cheong J., Balhorn M. E., Nagoshi C. T. (2001). A social learning perspective: A model of parenting styles, self-regulation, perceived drinking control, and alcohol use and problems. Alcoholism: Clinical and Experimental Research.

[B76-behavsci-16-01342] Patock-Peckham J. A., Corbin W. R. (2023). Impaired control over drinking predicts changes in alcohol-related consequences over and above alcohol use and facets of impulsivity. Addictive Behaviors.

[B77-behavsci-16-01342] Patock-Peckham J. A., Corbin W. R., Smyth H., Canning J. R., Ruof A., Williams J. (2022). Effects of stress, alcohol prime dose, and sex on ad libitum drinking. Psychology of Addictive Behaviors.

[B78-behavsci-16-01342] Patock-Peckham J. A., King K. M., Morgan-Lopez A. A., Ulloa E. C., Filson Moses J. M. (2011). Gender-specific mediational links between parenting styles, parental monitoring, impulsiveness, drinking control, and alcohol-related problems. Journal of Studies on Alcohol and Drugs.

[B79-behavsci-16-01342] Peltier M. R., Verplaetse T. L., Mineur Y. S., Petrakis I. L., Cosgrove K. P., Picciotto M. R., McKee S. A. (2019). Sex differences in stress-related alcohol use. Neurobiology of Stress.

[B80-behavsci-16-01342] Pilar M. R., Eyler A. A., Moreland-Russell S., Brownson R. C. (2020). Actual causes of death in relation to media, policy, and funding attention: Examining public health priorities. Frontiers in Public Health.

[B81-behavsci-16-01342] Pinto R., Correia L., Maia Â. (2014). Assessing the reliability of retrospective reports of adverse childhood experiences among adolescents with documented childhood maltreatment. Journal of Family Violence.

[B82-behavsci-16-01342] Portillo C., Adinoff B., Spencer C. T., Field C. A. (2023). The association of allostasis with alcohol use: A case–control study in males with and without alcohol use disorder. Alcohol.

[B83-behavsci-16-01342] Poznyak V., Rekve D. (2018). Global status report on alcohol and health 2018.

[B84-behavsci-16-01342] Pratt W. M., Davidson D. (2009). Role of the HPA Axis and the A118G polymorphism of the μ-opioid receptor in stress-induced drinking behavior. Alcohol and Alcoholism.

[B85-behavsci-16-01342] Ramchandani V. A., Stangl B. L., Blaine S. K., Plawecki M. H., Schwandt M. L., Kwako L. E., Sinha R., Cyders M. A., O’Connor S., Zakhari S. (2018). Stress vulnerability and alcohol use and consequences: From human laboratory studies to clinical outcomes. Alcohol.

[B86-behavsci-16-01342] Reck A., Sweet L. H., Geier C., Kogan S. M., Cui Z., Oshri A. (2024). Food insecurity and adolescent impulsivity: The mediating role of functional connectivity in the context of family flexibility. Developmental Science.

[B87-behavsci-16-01342] Reiss S., Peterson R. A., Gursky D. M., McNally R. J. (1986). Anxiety sensitivity, anxiety frequency and the prediction of fearfulness. Behaviour Research and Therapy.

[B88-behavsci-16-01342] Rubio G., Gasparyan A., Duque A., García-Gutiérrez M. S., Navarrete F., Navarro D., Manzanares J. (2025). Emotional processing and maltreatment during childhood as factors of vulnerability to alcohol abuse in young adults. International Journal of Mental Health and Addiction.

[B89-behavsci-16-01342] Sarason I. G., Johnson J. H., Siegel J. M. (1978). Assessing the impact of life changes: Development of the Life Experiences Survey. Journal of Consulting and Clinical Psychology.

[B90-behavsci-16-01342] SAS Institute Inc. (2025). SAS/STAT^®^ 9.4 user’s guide.

[B91-behavsci-16-01342] Sayette M. A., Fairbairn C. E., Creswell K. G., Kopetz C. E., Lejuez W. (2016). Alcohol and emotion: The importance of social context. Addiction: A social psychological perspective.

[B92-behavsci-16-01342] Shin S. H., Miller D. P., Teicher M. H. (2013). Exposure to childhood neglect and physical abuse and developmental trajectories of heavy episodic drinking from early adolescence into young adulthood. Drug and Alcohol Dependence.

[B93-behavsci-16-01342] Sinha R. (2008). Chronic stress, drug use, and vulnerability to addiction. Annals of the New York Academy of Sciences.

[B94-behavsci-16-01342] Sinha R. (2009). Modeling stress and drug craving in the laboratory: Implications for addiction treatment development. Addiction Biology.

[B95-behavsci-16-01342] Sinha R. (2022). Alcohol’s negative emotional side: The role of stress neurobiology in alcohol use disorder. Alcohol Research: Current Reviews.

[B96-behavsci-16-01342] Sinha R. (2024). Stress and substance use disorders: Risk, relapse, and treatment outcomes. The Journal of Clinical Investigation.

[B97-behavsci-16-01342] Snider S. E., Mellis A. M., Poe L. M., Kocher M. A., Turner J. K., Bickel W. K. (2020). Reinforcer pathology: Narrative of hurricane-associated loss increases delay discounting, demand, and consumption of highly palatable snacks in the obese. Psychology of Addictive Behaviors.

[B98-behavsci-16-01342] Sobsey D., Randall W., Parrila R. K. (1997). Gender differences in abused children with and without disabilities. Child Abuse & Neglect.

[B99-behavsci-16-01342] Spielberger C. D., Gorsuch R. L., Lushene R., Vagg P., Jacobs G. A. (1983). Manual for the state-trait anxiety inventory.

[B100-behavsci-16-01342] Substance Abuse and Mental Health Services Administration (SAMHSA) (2023). National survey on drug use and health. Table 5.9A—Alcohol use disorder in past year: Among people aged 12 or older; by age group and demographic characteristics, numbers in thousands, 2022 and 2023 *[Data Table]*.

[B101-behavsci-16-01342] Sze Y. Y., Stein J. S., Bickel W. K., Paluch R. A., Epstein L. H. (2017). Bleak present, bright future: Online episodic future thinking, scarcity, delay discounting, and food demand. Clinical Psychological Science.

[B102-behavsci-16-01342] Takahashi T., Ikeda K., Hasegawa T. (2007). A hyperbolic decay of subjective probability of obtaining delayed rewards. Behavioral and Brain Functions.

[B103-behavsci-16-01342] Thomas S. E., Bacon A. K., Randall P. K., Brady K. T., See R. E. (2011). An acute psychosocial stressor increases drinking in non-treatment-seeking alcoholics. Psychopharmacology.

[B104-behavsci-16-01342] Vandermiden J., Hamby S., Ferdon C. D., Kacha-Ochana A., Merrick M., Simon T. R., Finkelhor D., Turner H. (2019). Rates of neglect in a national sample: Child and family characteristics and psychological impact. Child Abuse & Neglect.

[B105-behavsci-16-01342] Vanderveldt A., Green L., Myerson J. (2015). Discounting of monetary rewards that are both delayed and probabilistic: Delay and probability combine multiplicatively, not additively. Journal of Experimental Psychology: Learning, Memory, and Cognition.

[B106-behavsci-16-01342] Vaughan C. L., Stangl B. L., Schwandt M. L., Corey K. M., Hendershot C. S., Ramchandani V. A. (2019). The relationship between impaired control, impulsivity, and alcohol self-administration in nondependent drinkers. Experimental and Clinical Psychopharmacology.

[B107-behavsci-16-01342] Vergés A., Lee M. R., Martin C. S., Trull T. J., Martens M. P., Wood P. K., Sher K. J. (2021). Not all symptoms of alcohol dependence are developmentally equivalent: Implications for the false-positives problem. Psychology of Addictive Behaviors.

[B108-behavsci-16-01342] Vuchinich R. E., Heather N. (2003). Choice, behavioral economics, and addiction.

[B109-behavsci-16-01342] Wagner J., Bell A. S., Jung J., Lohoff F. W., Mueller S., Heilig M. (2023). The genetics of alcohol use disorder. Alcohol and alcohol-related diseases.

[B110-behavsci-16-01342] Wang A. Y., Vereschagin M., Richardson C. G., Xie H., Hudec K. L., Munthali R. J., Munro L., Leung C., Kessler R. C., Vigo D. V. (2023). Evaluating the effectiveness of a codeveloped e-mental health intervention for university students: Protocol for a randomized controlled trial. JMIR Research Protocols.

[B111-behavsci-16-01342] Weckesser L., Pilhatsch M., Muehlhahn M., Endraß T., Miller R. (2025). A meta-analysis on the relation between acute stress exposure, alcohol consumption and cortisol levels in individuals with a personal, familial or no alcohol use disorder. Translational Psychiatry.

[B112-behavsci-16-01342] White A. M. (2020). Gender differences in the epidemiology of alcohol use and related harms in the United States. Alcohol Research: Current Reviews.

[B113-behavsci-16-01342] White J. D., Bierut L. J. (2023). Alcohol consumption and alcohol use disorder: Exposing an increasingly shared genetic architecture. American Journal of Psychiatry.

[B114-behavsci-16-01342] Whiteside S. P., Lynam D. R. (2001). The Five Factor Model and impulsivity: Using a structural model of personality to understand impulsivity. Personality and Individual Differences.

[B115-behavsci-16-01342] Widom C. S., Czaja S. J., DuMont K. A. (2015). Intergenerational transmission of child abuse and neglect: Real or detection bias?. Science.

[B116-behavsci-16-01342] Wood M. D., Nagoshi C. T., Dennis D. A. (1992). Alcohol norms and expectations as predictors of alcohol use and problems in a college student sample. The American Journal of Drug and Alcohol Abuse.

[B117-behavsci-16-01342] Yeh Y.-H., Myerson J., Green L. (2021). Delay discounting, cognitive ability, and personality: What matters?. Psychonomic Bulletin & Review.

[B118-behavsci-16-01342] Yu J., Haynie D. L., Gilman S. E. (2024). Patterns of adverse childhood experiences and neurocognitive development. JAMA Pediatrics.

[B119-behavsci-16-01342] Zhao Y., Potenza M. N., Tapert S. F., Paulus M. P. (2023). Neural correlates of negative life events and their relationships with alcohol and cannabis use initiation. Dialogues in Clinical Neuroscience.

